# Decoding Handwriting Trajectories from Intracortical Brain Signals for Brain‐to‐Text Communication

**DOI:** 10.1002/advs.202505492

**Published:** 2025-07-28

**Authors:** Guangxiang Xu, Zebin Wang, Kedi Xu, Junming Zhu, Jianmin Zhang, Yueming Wang, Yaoyao Hao

**Affiliations:** ^1^ The State Key Lab of Brain‐Machine Intelligence Zhejiang University Hangzhou 311121 China; ^2^ Nanhu Brain‐computer Interface Institute Hangzhou 311121 China; ^3^ Department of Biomedical Engineering Zhejiang University Hangzhou 310027 China; ^4^ Department of Neurosurgery Second Affiliated Hospital Zhejiang University School of Medicine Hangzhou 310009 China; ^5^ College of Computer Science and Technology Zhejiang University Hangzhou 310027 China

**Keywords:** brain‐computer interface, brain‐to‐text, handwriting, shape and time distortion, trajectory fitting

## Abstract

The potential to decode handwriting trajectories from brain signals has yet to be fully explored in clinical brain‐computer interfaces (BCIs). Here, intracortical neural signals are recorded from a paralyzed individual during attempted handwriting of complex characters. An innovative decoding framework is introduced to address both shape and temporal distortions between neural activity and movement, effectively resolving the misalignment issue commonly encountered in clinical BCIs due to the lack of accurate movement labels. The results demonstrated the reconstruction of highly accurate and human‐recognizable handwriting trajectories, significantly outperforming conventional methods. Furthermore, the new framework enabled effective multi‐day data fusion, leading to additional improvements in trajectory quality. By employing a dynamic time warping approach to translate trajectories into text, a recognition rate up to 91.1% is achieved within a 1000‐character database. Additionally, the framework is applied to reconstruct single‐trial trajectories of English letters using a previously published dataset, achieving similarly high recognition rates. Collectively, these findings present a novel BCI decoding scheme capable of accurately reconstructing handwriting trajectories, demonstrating its applicability to both alphabetic and logographic brain‐to‐text translation. This approach has the potential to revolutionize communication for individuals with motor impairments by enabling accurate brain‐to‐text translation across diverse languages.

## Graphical abstract

By developing a novel framework that optimizes both shape and temporal loss during decoder training, the authors successfully reconstruct human‐recognizable handwriting trajectories from intracortical neural signals for both Chinese characters and English letters, effectively resolving the temporal misalignment problem in clinical BCIs, thereby establishing a robust brain‐to‐text communication plateform adaptable to diverse writing systems.

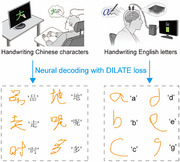

## 1 Introduction

Over the past two decades, intracortical brain‐computer interfaces (BCIs) have emerged as a revolutionary tool that enables direct communication between the human brain and external devices.^[^ ^1^, ^2^, ^3^, ^4^, ^5^, ^6^, ^7^, ^8^, ^9^ ^]^ The intracortical BCIs have opened new avenues for individuals with motor deficits to interact more effectively with their surroundings, leading to extensive clinical trials being conducted worldwide.^[^ ^10^ ^]^

The incorporation of handwriting paradigm into BCIs marks a substantial advancement in the field, enabling users to convert attempted handwriting movements into textual output. A seminal work in this domain by Willett et al.^[^ ^7^ ^]^ demonstrated the feasibility of classifying neural activity into English letters with an impressive 94.1% raw classification accuracy within a 30‐character scope. This process was then efficiently transcribed into text at a rate of ≈90 characters per minute. These advancements highlighted the exceptional capabilities of a high‐performance BCI paradigm^[^ ^7^ ^]^ which was explored recently with scalp recordings.^[^ ^11^, ^12^, ^13^, ^14^, ^15^ ^]^ However, the classification‐based decoding scheme utilized in the study was specifically designed for Latin‐based languages, which require discrimination of only a few dozen letters to form text. In contrast, non‐Latin languages, such as Chinese, demand classification of thousands of distinct characters – a challenge that is currently beyond the capabilities of neural signal‐based classification for BCIs. Therefore, there is an urgent need for an innovative decoding scheme that could reconstruct the trajectories of handwritings, instead of just identities, to realize a brain‐to‐text system for any written characters. In this context, the overall shape of the handwriting trajectories is far more critical than minimizing the overall error. This underscores the importance of developing a decoding approach that prioritizes the preservation of the trajectory's overall shape, a feature essential for accurate text translation across diverse languages.

Trajectory fitting is a well‐established technique in BCIs, which translates movement intention into continuous motion of computer cursors or robotic devices.^[^ ^16^, ^17^ ^]^ Most of the prior studies have employed mean squared error (MSE) loss to optimize the trajectory fitting algorithms, aiming to develop optimal decoders.^[^ ^18^, ^19^ ^]^ However, MSE has its own inherent limitations when assessing predictions, particularly in scenarios with sharp variations.^[^ ^20^, ^21^, ^22^ ^]^ While this limitation is not critical in simple trajectory tasks, such as center‐out movements, it becomes pronounced in complex cases like cursive moving^[^ ^23^, ^24^ ^]^ or handwritings,^[^ ^7^, ^12^ ^]^ where precise path following is required. Furthermore, in clinical BCI applications, there is often an absence of labeled movement to training the decoder, due to the motor impairment of the subject. In this case, an initial imaginary or attempting movement process was conducted following a guidance for the subject.^[^ ^7^, ^8^ ^]^ These guidance movements are taken as the surrogate label to train an initial decoder, which is then further optimized during subsequent online control experiments. However, the subject's thoughts may either precede or lag the guidance movements, leading to misalignment which results in less precise decoding models. The MSE method enforces a point‐to‐point alignment and distributes the loss across the entire trial to achieve a global minimum, which is not well‐suited for situations with misalignment.^[^ ^25^ ^]^ Therefore, there is a clear demand for a loss function capable of accounting for alignment with sharp variations to ensure accurate decoding in practical BCI settings.

In this study, we acquired intracortical neural signals from a patient during attempted handwritings, with the goal to reconstruct the very trajectories of the handwritings and recognizing them as standard text. We introduced a novel decoding framework that specifically addressed the misalignment issue in the paradigm and reconstructed human‐recognizable handwriting trajectories. Comparing with conventional method, novel framework achieved high‐fidelity of trajectory reconstruction and better recognition rate in both single‐day and multi‐day fusion decoding. These advancements hold profound implications for the field of BCI, offering a promising new pathway toward more accurate handwriting‐based communication for a wide range of users with motor impairments.

## 2 Results

**Figure 1:**
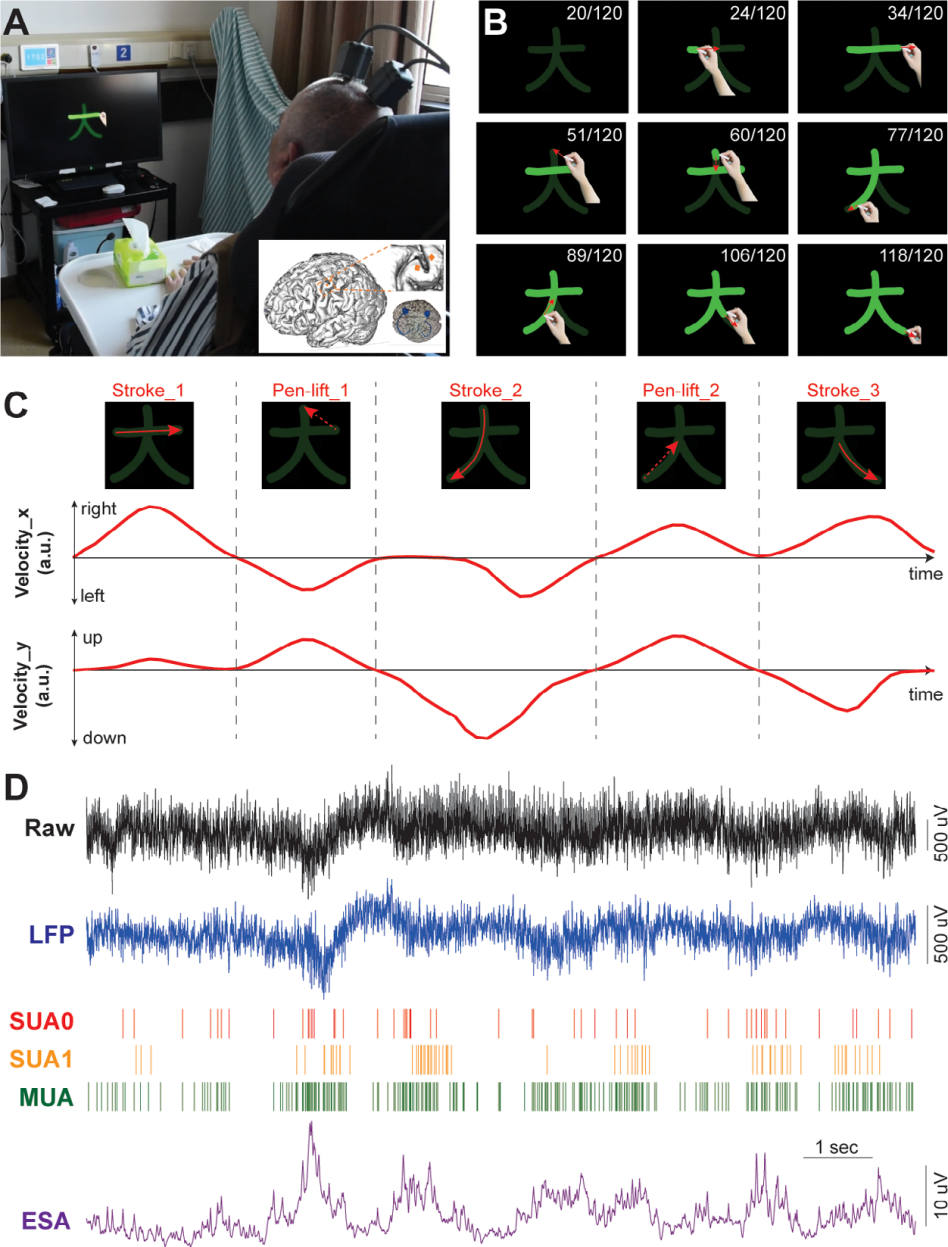
Experimental setup and neural signal recording. A) The subject with recording cables connected was asked to attempt to handwrite following the animation shown on the screen. Inset: illustration of implantation position for the two Utah arrays (orange) and two connectors (blue) in the left hemisphere. B) Example frames of the animation video writing a Chinese character “大”. The red arrow indicates the moving direction (not shown in the experiment). The number in the upper right indicates the current frame/total frame number (not shown in the experiment). C) Velocity profile (red lines) in x and y directions for the character “大”. The dashed lines separate the three strokes and two pen lifts, which are represented by red solid and dashed arrows, respectively. D) Example of neural signals when handwriting a character, including raw signal (30k sampling, 0.3–7500 Hz) and other processed signal features, like LFP, SUA, MUA, and ESA.

We surgically implanted two Utah arrays into the left motor cortex of a patient, specifically targeting the region surrounding the hand “knob” area (**Figure** **1**A inset). The patient, a right‐handed individual in his 70′s, had experienced a C4‐level spinal cord injury resulting in total sensory and motor loss below the shoulders. During experimental sessions, the patient was instructed to attempt to handwrite characters with his right‐hand using chalk on a blackboard, following a video displayed on a screen (Figure 1A). The video presented handwriting sequences of strokes and pen lifts—representing the inter‐stroke movements—of a single character at an overall consistent speed (Figure 1B). Figure 1C illustrates the smoothed velocity profiles in the *x*‐ and *y*‐direction during the writing of a character that comprised of three strokes and two pen lifts, with each stroke or pen lift exhibiting a bell‐shaped velocity curve.

We asked the subject to attempt to write 180 unique Chinese characters across 6 sessions (30 characters per session, with each character repeated three times). These characters are commonly used in daily life, with an average of 7.06 ± 2.78 strokes per character. We recorded raw neural signals during the handwriting process. From these signals, we extracted neural features from both low and high‐frequency bands (Figure 1D), encompassing a range of measurements such as local field potential (LFP), single and multiple unit activity (SUA and MUA), entire spike activity (ESA, a continuous signal derived from high‐pass filtering, rectification, and low‐pass filtering of raw neural data^[^ ^18^ ^]^), etc. (see Experimental Section). We demonstrated that the neural activity patterns for each character were highly distinct; an SVM classifier based on SUA achieved nearly perfect discrimination accuracy of 98.2% ± 2.29% among the 30‐characters in each session. The classification accuracy was comparable to previous classification results for handwriting of 30 English letters and punctuations.^[^ ^7^ ^]^

### 2.1 Handwriting Trajectory Decoding and Text Translation

**Figure 2:**
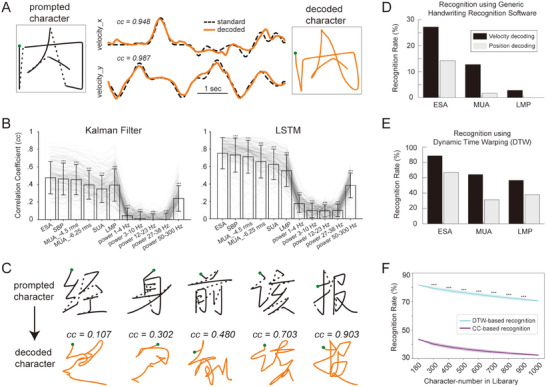
Handwriting trajectory fitting with MSE loss. A) Example of a prompted Chinese character “内” (solid for strokes and dash line for pen lifts, left panel) and the decoded trajectory (orange, right panel). The corresponding velocity profiles in x and y directions were showed in the middle panel. Green dots indicate the start of the handwriting. B) The fitting correlation coefficients (CC) of the 180 characters for Kalman Filter (left panel) and LSTM (right panel) decoder with various kinds of neural signals. See text for the abbreviation. Asterisk (^***^) indicates significant difference between ESA and other signals (paired signed‐rank test, *p* < 0.001). (C) Decoded trajactory examples for five characters (“经,” “身,” “前,” “该,” “报”) with fitting CC ranging from 0.1 to 0.9. D) Recognition rate of the 180 decoded characters using a generic handwriting character recognition API (from teshuzi.com, v1). The decoding results for LMP, MUA, and ESA were illustrated. Decoding scheme for both velocity (black bar) and position (gray bar) was tested. E) Same as (D) but for dynamic time warping (DTW) based recognition method which calculates similarity between the decoded trajectories and the standard Chinese character trajectories (180 characters in library). F) DTW‐ and CC‐based recognition rate as a function of character number in the library. Shade indicates SD from 1000 times resampling and ^***^ means significant difference (Mann–Whitney U‐test, *p* < 0.001).

To realize brain‐to‐text translation, we fit the neural activities into the velocity of the handwriting, reconstructed the trajectory by performing an integration along the path (**Figure** **2**A), and then recognized the trajectories as standard texts using machine learning approaches (detailed pipeline in Figure S1A, Supporting Information). We trained both linear Kalman filter and nonlinear long short‐term memory (LSTM) network using leave‐one‐character‐out cross‐validation for trajectory fitting. The goal of these two decoders was to minimize the mean square error (MSE) between the prompted writing velocity and the decoded velocity. The decoding results of various types of neural features, quantified as correlation coefficient (CC), were presented in Figure 2B. Across all scenarios, the LSTM demonstrated superior fitting outcomes compared to the Kalman filter. Notably, ESA yielded significantly better results than all other signal features, with an average CC of 0.753 ± 0.18. To provide a qualitative illustration of how the reconstructed trajectories varied with different CC values, we showcased five example reconstructions in Figure 2C, with CC values ranging from 0.1 to 0.9. Generally, a reconstruction with a CC exceeding 0.5 would result in a human recognizable shape. However, quantitative results were required to assess the quality of reconstructed trajectories.

To objectively assess whether the decoded trajectories could be recognized as legible text, we initially utilized a generic handwriting recognition software to discern the continuous trajectory for each character. In this scenario, we employed neural features—ESA, SUA, and local motor potential (LMP, moving average of LFP with 50 ms window)^[^ ^26^ ^]^—for both velocity decoding (integrated to derive position) and direct position decoding using an LSTM decoder (Figure 2D). Velocity decoding using the ESA feature yielded the highest recognition rate; however, only around a quarter (27.2%) of the trajectories could be recognized as correct Chinese characters (Figure 2D). This was not a surprise because the decoded trajectory for each character was essentially a single continuous stroke, which significantly deviates from conventional stroke‐by‐stroke handwriting patterns that were used to train the generic handwriting recognition software.

To improve trajectory recognition accuracy, we developed an alternative method that identifies the standard character whose speed profile most closely matches the decoded trajectories. The underlying concept was that each character would generate a unique and distinctive speed profile identifier along the writing process. To that end, we first built a library that encompassed the speed profiles for writing the standard 180 characters. Then each decoded trajectory was z‐score normalized and matched with the most similar standard character using the dynamic time warping (DTW) algorithm (see Experimental Section). Velocity decoding with ESA feature achieved the highest recognition rate, and ≈81.7% of the trajectories could be correctly recognized within 180 characters (Figure 2E). Given that the speed profiles were highly unique for each character, only a slight decrease in recognition rate to 70.6% was observed when the library expanded to 1000 characters (Figure 2F). Compared to the CC‐based method, DTW accommodates temporal variations by allowing sequences to exhibit time‐axis delays or stretches (Figure S1B,C, Supporting Information), which significantly improved recognition rates (Figure 2F). This suggested that the recognition method was sufficiently robust for recognizing a large number of characters.

### 2.2 Novel Decoding Framework with Shape and Time Distortion

One possible issue with clinical BCI paradigm was that the patient may not have the exactly same speed profile as that of the guiding video, i.e., the neural activity and the label velocity profile were misaligned. This problem is commonly encountered in clinical experiments with subjects who had lost their motor ability. Critically, the misalignment was not uniform across a single trial, making the problem more severe. The MSE loss of neural decoder employed previously overlooked this misalignment, which could train suboptimal decoders that affected the decoding performance. Therefore, we employed a recently introduced distortion loss including shape and time, referred as DILATE,^[^ ^20^ ^]^ to specifically address this prevalent problem in practical BCIs. Since backpropagation of the loss was required to improve neural decoding performance, we exclusively employed the LSTM decoder to evaluate the loss functions (DILATE vs MSE). Unless otherwise stated, all subsequent decoding results were obtained using velocity decoding with ESA feature.

**Figure 3:**
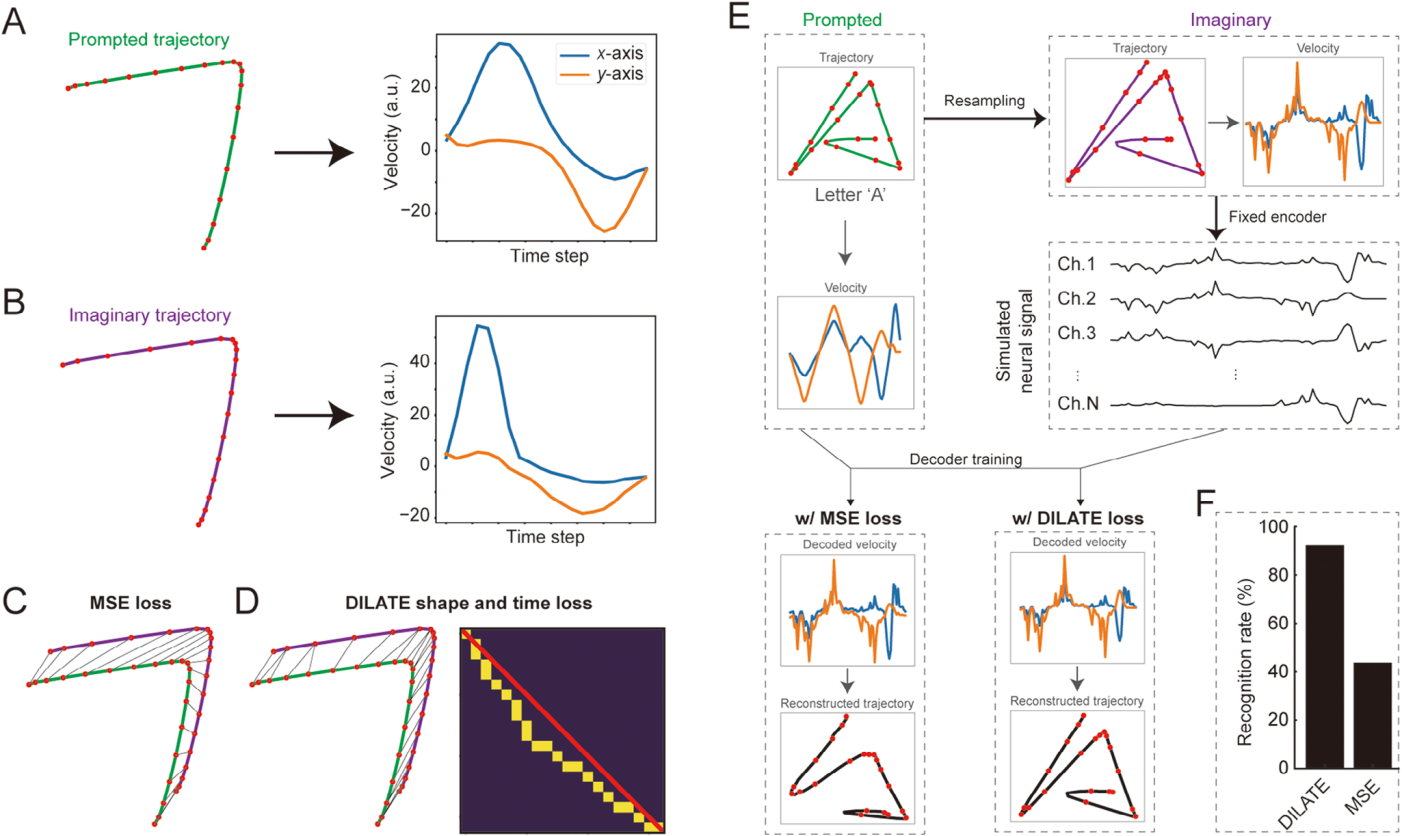
DILATE versus MSE loss for trajectory fitting. A) Example of a prompted trajectory (dots indicate positions at equal time intervals) for handwriting experiment and its corresponding velocity profiles in *x*‐ and *y*‐axis. B) Same as (A) but for the actual trajectory adopted by the subject. Note the sampling dots have different positions from (A) and thus velocity profiles are slightly different. C) Schematic diagram for calculating MSE loss. The line between the prompted and actual trajectory indicates the point‐to‐point alignment of the MSE loss. D) Schematic diagram for calculating DILATE loss. The line between the prompted and actual trajectory (left panel) and the sequence of yellow dots (right panel) indicates the shape and time alignment in DILATE loss. Shape loss calculates the distance between points, and time loss calculates the offset of the aligned paths with respect to one‐by‐one alignment (red line). E) Schematic diagram of decoding simulation experiment. First, the prompted velocities were randomly resampled to obtain the “actual” velocities. Then, a fixed linear encoder is used to encode the actual velocities into simulated neural signals. Finally, based on the simulated neural signals and prompted velocities, the decoder is trained using MSE loss and DILATE loss respectively to obtain the decoded velocities to reconstruct the trajectories. F) Recognition rate of the decoded trajectories (from letter “A” to “Z,” 10 resamples per letter) in the simulated experiments using a generic handwritten character recognition software.

When the prompted trajectory (**Figure** **3**A) and “actual” trajectory executed by the subject (Figure 3B) are not aligned in velocity but have the same shape, the MSE loss still calculates the loss value based on a one‐to‐one matching as shown in Figure 3C. DILATE, on the other hand, was designed to stretch or compress time‐series data point‐by‐point to make them as aligned as possible. It consists of shape loss and time loss. The goal of shape loss, which is essentially a soft DTW, is to find the optimal time alignment path that minimizes the overall distance between two time series (Figure 3D, left). The soft DTW replaces the hard minimization operation in DTW by introducing a differentiable soft minimization operation, which makes the temporal alignment path smoother instead of enforcing a strict match between time points. The time loss, on the other hand, is to constrain that the optimal time alignment paths between prediction and goal do not deviate too much from the one‐to‐one matching alignment paths (Figure 3D, right).

To demonstrate the advantage of the DILATE loss over the MSE loss when the signals are not aligned with the labels, we designed a simulated decoding experiment (Figure 3E). In a database with handwriting trajectories of 26 English letters, we randomly resampled the velocity profiles for each letter under the condition that the reconstructed shape was guaranteed to be unchanged. Then a fixed linear encoder model transferred the resampled velocities into the simulated neural signals, which are, in principle, not aligned with the original velocities. We finally trained the same decoder using either MSE or DILATE loss and evaluated the reconstructed trajectories separately. The recognition rate using a generic handwriting recognition software for MSE decoding was 43.8%, whereas decoding with DILATE resulted in a recognition rate of 92.3% (Figure 3F). We also did the same simulation with the180 Chinese characters used for the patient and the results showed similar recognition rates of 100% and 16.7% for DILATE and MSE loss, respectively. This comparison demonstrated that DILATE effectively addresses the misalignment issue, enabling the construction of accurate decoders.

To illustrate the reason why DILATE outperformed MSE under conditions of misalignment, we conducted a simulation in a simpler scenario (Figure S2, Supporting Information). Assuming that the actual velocity and the prompted velocity *y* were identical copies but only with an overall delay in time (Figure S2A, Supporting Information), and the neural signal *x* was identical (i.e., linear) to the actual velocity. We assumed a linear decoder (*y = ax + b*) to map the neural signal to the true velocity. When using MSE to train the decoder, the smallest loss value was found at *a* = 0 and *b* = 1, which was basically a flat line (Figure S2B, Supporting Information). In contrast, DILATE minimized at *a* = 1 and *b* = 0, which was an identical function, i.e., the shape was retained regardless of the delay (Figure S2C, Supporting Information). This result indicates that DILATE loss valued the overall shape matching (after distortion) instead of pursuing overall one‐to‐one error minimization as in MSE, which is exactly what we need in the handwriting BCIs where accurate path reconstruction is critical.

### 2.3 Handwriting Trajectory Decoding with New Framework

**Figure 4:**
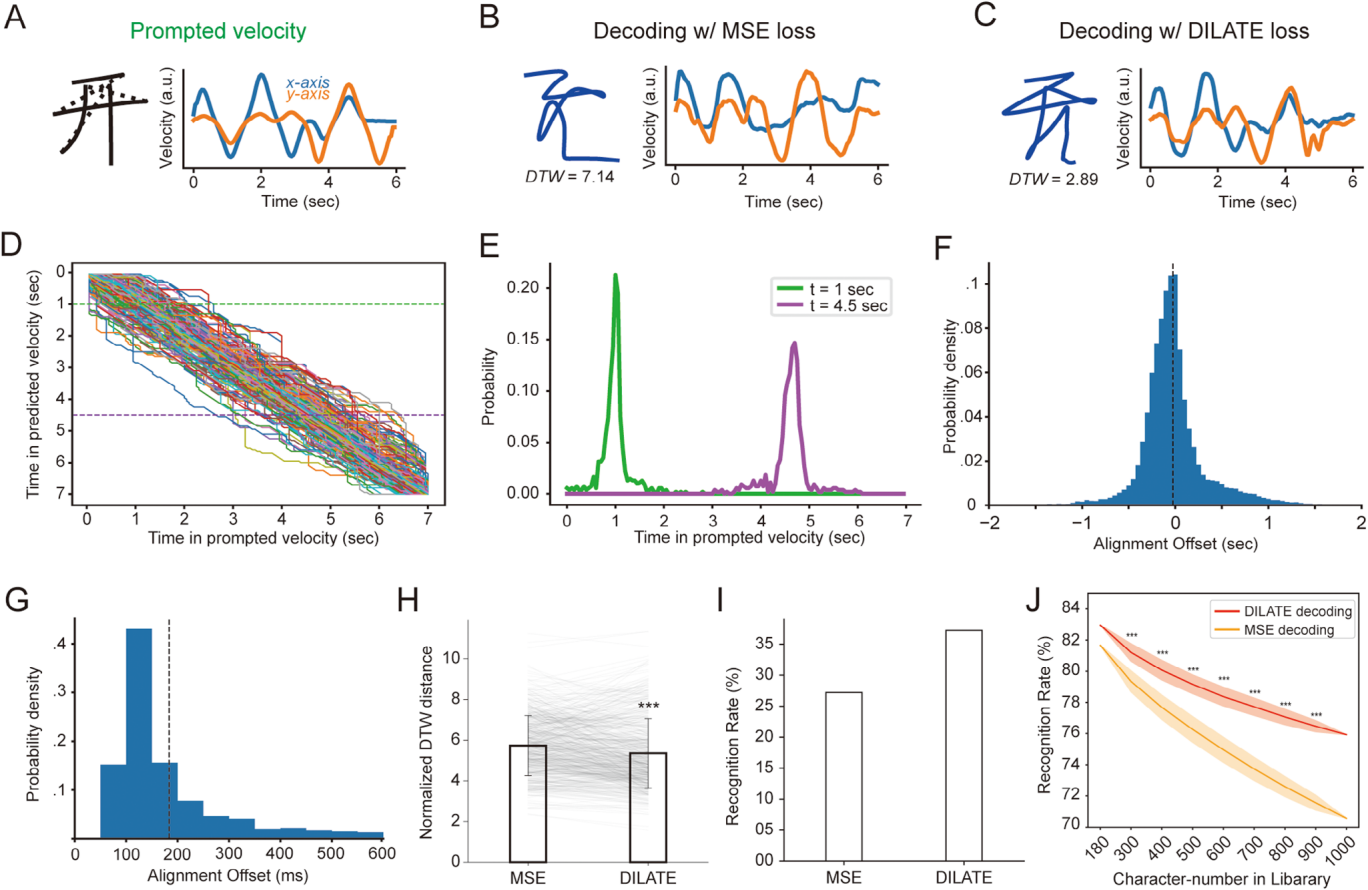
Trajectory fitting with DILATE loss. A) Example of a standard Chinese character “开” (solid for strokes and dash for pen lifts, left panel) and the corresponding velocity profiles in *x*‐ and *y*‐axis (right panel). B,C) Same as (A) but for decoding result with MSE (B) and DILATE (C) loss. The DTW distances between decoded and prompted trajectories are indicated. D) The alignment paths of DILATE for all the decoded and prompted velocities in all sessions. Each line represents one character in a trial. E) Probability distribution of time steps that are aligned with the decoded velocity at times of 1.0 and 4.5 s, i.e., two dashed lines in (D). F) Histogram of the alignment offsets for all trials. G) Histogram of standard deviation of overall alignment offsets for predicted and label velocities for all trials. H) Normalized DTW distance between all the decoded and prompted characters for MSE loss and DILATE loss (^***^, *p* < 0.001, paired signed‐rank test). I) Recognition rate of all the decoded trajectories using a generic handwritten recognition software for MSE and DILATE decoding. J) DTW‐based recognition rate for DILATA versus MSE decoding as a function of character number in the library. Shade indicates SD from 1000 times resampling and ^***^ means significant difference (Mann–Whitney U‐test, *p* < 0.001).

We next attempted to use the DILATE loss to train the decoder to fit the velocity of the handwriting of Chinese characters from the neural signal and reconstruct the trajectories. We first searched the two hyperparameter tradeoff *α* (0.1–0.9) and smoothing factor γ (0.0001–1) in the DILATE, and found that these two parameters had little effect on the decoding performance (Figure S2D, Supporting Information); the final choice of parameters was *α* = 0.5 and γ = 0.001 throughout the study. As the example character showed in **Figure** **4**A, LSTM‐based decoding with the DILATE loss produced trajectories that more closely resembled the target in both shape and stroke dynamics (Figure 4B), and exhibited a smaller DTW distance—a metric quantifying similarity between time series with local temporal variations (see Experimental Section)—compared to MSE‐based decoding (Figure 4C). Additional reconstructed trajectory examples are provided in Figure S3 (Supporting Information). Beyond LSTM, we evaluated alternative deep learning architectures, including standard recurrent neural networks (SRNNs), gated recurrent units (GRUs), temporal convolutional networks (TCNs), and Transformers (parameters shown in Table S1, Supporting Information). Results demonstrated that LSTMs consistently achieved high and stable performance across both MSE and DILATE loss functions (Figure S4, Supporting Information), and were adopted as the primary architecture for subsequent analyses.

To reveal the actual alignment in DILATE, the normalized alignment paths in the algorithm for each trial (i.e., character) were plotted in Figure 4D. The paths formed a thick cloud along the diagonal, which indicated that the optimal alignment was mostly time‐distorted; if not distorted, the lines will be exactly diagonal. The probability density plots of two slices at 1.0 and 4.5 s (Figure 4E) showed that the main alignment offsets were ranging from ‐500 to 500 ms. To quantitatively measure the alignment offset, we calculated the single‐point offsets for each character and plotted the histograms in Figure 4F. The averaged alignment offset was ‐24.15 ± 356.07 ms, which indicated that the overall alignment offset is small but the local alignment offset fluctuates widely. We also calculated the standard deviation (SD) of the single‐point offsets within each trial (Figure 4G), revealing a wide SD of 183.62 ± 122.39 ms. These results indicate that the alignment in each trial was nonuniform and could be leading or lagging at different part of a trial.

Finally, we quantitatively compared the decoding results for DILATE versus MSE loss. The decoding results for all the 180 Chinese characters using DILATE were significantly better than those using MSE, with lower normalized DTW distance (5.35 ± 1.71 vs 5.73 ± 1.47, Figure 4H) and higher recognition rate with generic handwriting recognition software (37.2% vs 27.2%, Figure 4I). Using DTW‐based recognition, DILATE decoding achieved a significantly higher recognition rate compared to MSE‐based decoding across all library sizes (Figure 4J). We additionally evaluated the real‐time performance of the DILATE‐based decoding framework, demonstrating a sustained output rate of ≥ 250 Hz in a pseudo‐online experiment (Figure S5, Supporting Information), which surpasses the temporal requirements for most practical BCI applications. Figure S6 (Supporting Information) further demonstrated the robustness of DILATE‐based decoding to increasing noise levels. Confusion matrix analysis (Figure S7, Supporting Information) shows significantly shorter DTW‐distance with frequently confused recognitions in both MSE‐ and DILATE‐based decoding, indicating that the overall similar writing profiles lead to confusion. Therefore, we demonstrated that the DILATE loss accounted for optimal alignment at each time step and achieved better decoding performance.

### 2.4 Multi‐Day Data Fusion Enabled by the New Framework

**Figure 5:**
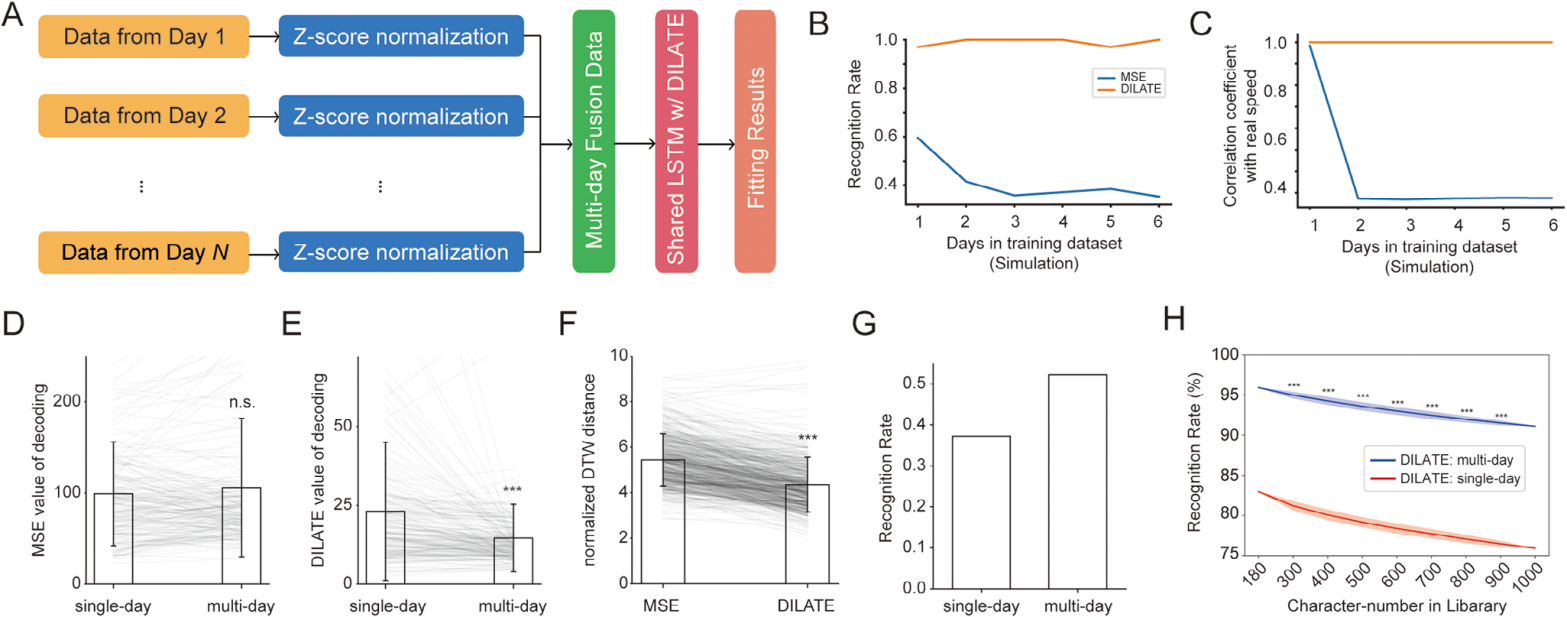
Multi‐day data fusion with DILATE loss. A) Schematic diagram of the multi‐day fusion decoding framework. Multi‐day neural data are mixed after z‐score normalization, and a shared LSTM network is then trained using the DILATE loss (or MSE loss) to obtain the fitting results. B,C) The recognition rate (B), correlation coefficient (C) in multi‐day simulation experiments (see text for details). D) MSE loss values of single‐day and multi‐day decoding results with MSE loss (*n.s*., no significance, *p* > 0.05, paired sign rank test). E) DILATE loss values of single‐day and multi‐day decoding results with DILATE loss (^***^, *p* < 0.001, paired signed‐rank test). F) Normalized DTW distance for multi‐day decoding results with MSE loss and DILATE loss (^***^, *p* < 0.001, paired signed‐rank test). G) Recognition rate using the generic handwritten recognition software for single‐day and multi‐day decoding with DILATE. H) DTW‐based recognition rate as a function of character number in the library for DILATE multi‐day and single‐day decoding. Shade indicates SD from 1000 times resampling and ^***^ means significant difference (Mann–Whitney U‐test, *p* < 0.001).

We then expanded the application of the DILATE loss to a multi‐day scenario, where a more extensive dataset was created by integrating data from multiple days to train a new decoder, with the expectation of achieving improved performance (**Figure** **5**A). However, due to factors such as shifts in neural activity and data quality control, it was not guaranteed that the new decoder would perform better with an increased training dataset. We hypothesized that the DILATE loss, with its ability to effectively align neural activity with the target, could generate higher‐quality of training data. Consequently, we anticipated that the decoding performance derived from multi‐day data could potentially outperform that of single‐day data.

To validate the hypothesis, we first conducted a simulation experiment akin to Figure 3E, but with multi‐day data. In the experiment, new data from up to 6 days were added to the first day's decoding. For each day, the velocities were subjected to random resampling to generate unaligned simulation signals. The DILATE decoding consistently sustained superior decoding performance, showing no sign of degradation as the number of days increased. In contrast, the MSE loss exhibited relatively inferior performance and eventually plateaued at a stable level (Figure 5B). We then quantified the similarity between the decoded velocity profiles with the resampled velocity, which represented the extent to which decoding speeds can still approach real speeds in the presence of label misalignment. For DILATE, the CC remained stable, whereas with the traditional MSE loss, there was a noticeable decline in similarity as the data in the training set increased (Figure 5C).

To evaluate DILATE on real‐word data, we normalized the neural feature (i.e., ESA) using z‐score for each channel and combined data from all 6 days (30 characters/day) for training a shared decoder (Figure 5A). In other words, each character now was decoded by decoders trained with 174 (29 × 6) other characters, instead of 29 characters in the single‐day case. When trained with MSE loss, the average MSE loss values of the multiday decoding result were not significantly different from those of single‐day decoding (105.83 ± 75.85 vs 99.03 ± 56.99, *p* > 0.05, Figure 5D). However, when trained with DILATE loss, the average DILATE loss values for the multi‐day decoding were significantly reduced compared to single‐day decoding (15.17 ± 11.14 vs 23.99 ± 22.76, *p* < 0.001, Figure 5E). We then employed the normalized DTW distance as a standard metric to assess the multi‐day fusion decoding outcomes with both loss functions. The decoding results achieved with DILATE were significantly superior than those of MSE (4.35 ± 1.20 vs 5.44 ± 1.15, *p* < 0.001, Figure 5F). Most notably, the multi‐day fusion decoding using the generic handwriting recognition software yielded a recognition rate of 52.2%, substantially higher than that of the single‐day decoding of 37.2% (Figure 5G). When the DTW‐based recognition method was used, the recognition rate for DILATE multi‐day decoding achieved 91.1% in a 1000‐character database, significantly higher than DILATE single‐day decoding across all library size (Figure 5H; Figure S8, Supporting Information). These results indicated that the DILATE loss effectively mitigated the challenges associated with the multi‐day data fusion problem in BCIs.

### 2.5 Handwriting Trajectory Decoding and Text Translation for English Letters

**Figure 6:**
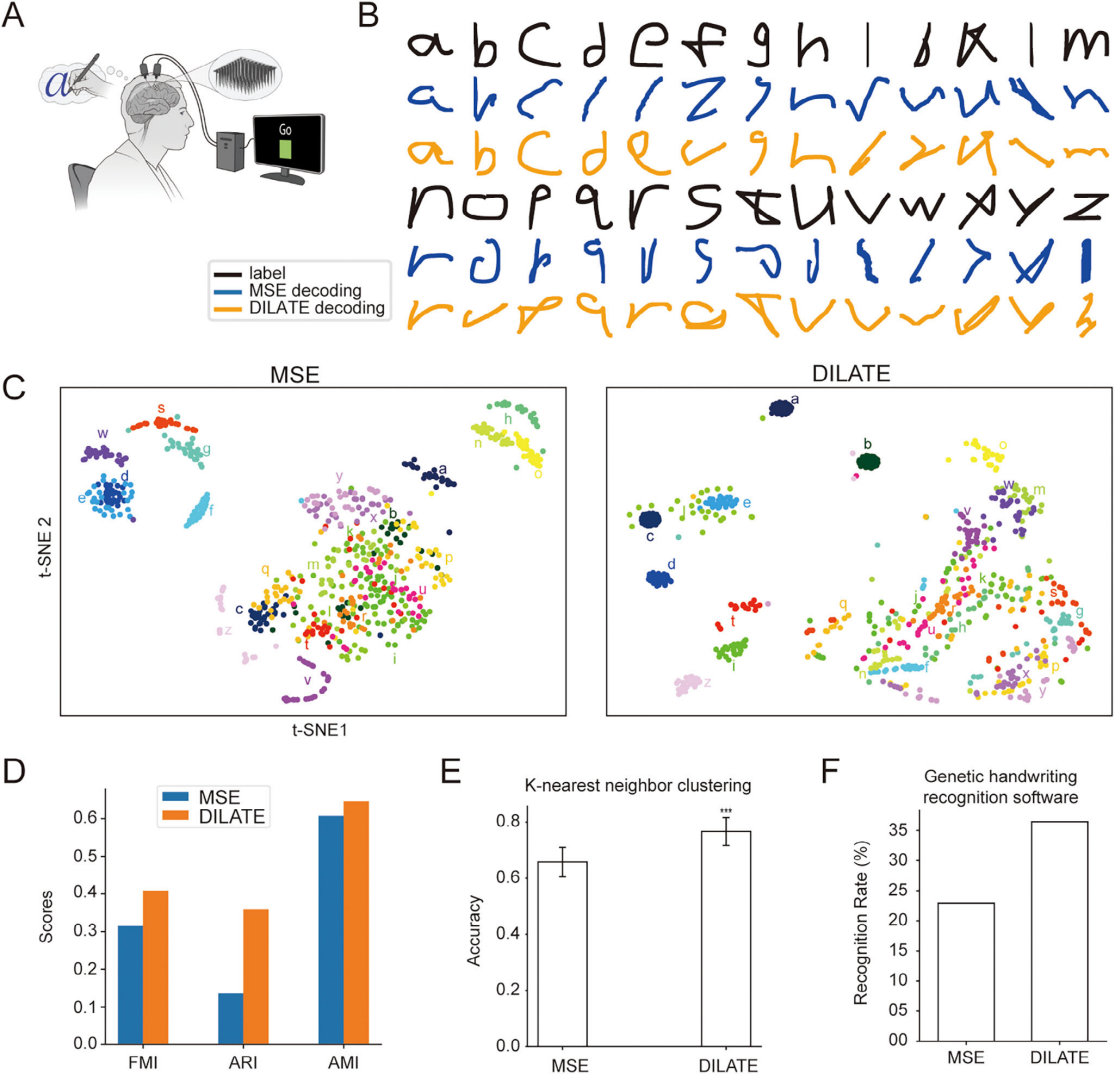
Trajectory fitting of English letters with DILATE loss. A) Schematic of the English letters handwriting experiments (Reproduced with permission.^[^ ^7^ ^]^ 2021, Springer Nature.). B) Example trajectories of all the letters reconstructed with MSE and DILATE loss. C) Visualization of t‐SNE dimensionality reduction for the decoding results with MSE loss (left panel) and DILATE loss (right panel). The clusters are labeled with letter annotations. D) Evaluation of the clustering with three metrics, FMI, ARI, and AMI, for MSE and DILATE decoding. E) Classification accuracy with k‐nearest neighbor for MSE and DILATE decoding (^***^, *p* < 0.001, *t*‐test). F) Recognition rate of the decoded letters using a generic handwritten character recognition software for MSE and DILATE decoding.

To further evaluate the capabilities of DILATE‐based decoding, we utilized a previously published dataset of English letter handwriting.^[^ ^7^ ^]^ In this dataset, intracortical neural activity was recorded from the motor cortex of a paralyzed subject who attempted to handwrite letters and symbols following a cue within a fixed interval (**Figure** **6**A). The main difference with our study was that there was an absence of video guidance for letter writing, which could result in more pronounced misalignment between the neural signals and handwriting movements. In their original study,^[^ ^7^ ^]^ the trajectory was reconstructed only with the fine‐aligned, trial‐averaged neural activity.

We used DILATE to investigate whether the neural signal could be automatically aligned at the single‐trial level, thereby improving the reconstructed trajectory. Figure 6B presents the example label trajectories for the letters that were required to copy for the patient, along with the decoded trajectories using the MSE and DILATE loss, respectively. In general, the decoding results using DILATE outperformed those of MSE, yielding more human‐recognizable letters.

We next quantitatively analyzed the decoding results. We first assessed the discriminability of the decoded results for both loss functions by applying t‐SNE downscaling followed by HDBSCAN clustering (Figure 6C). The decoding results using DILATE achieved better results in all three clustering evaluation metrics, including Fowlkes–Mallows Index (FMI), Adjusted Rand index (ARI), and Adjusted Mutual Information (AMI) (Figure 6D). We then employed the k‐nearest neighbor classification algorithm to categorize the decoding results for both loss functions. The classification results using DILATE achieved a correct rate of 76.61 ± 4.97%, which was significantly better than the 65.71 ± 5.25% obtained with MSE (Figure 6E). Finally, we evaluated the decoding results with the two loss functions by directly feeding the decoded trajectories to the generic handwriting recognition software. The decoding results using DILATE achieved a recognition rate of 36.47%, outperforming the 22.93% achieved with MSE (Figure 6F). These results demonstrated superior decoding performance of DILATE over MSE loss on both the Chinese character and English letter handwriting datasets.

## 3 Discussion

In this study, we recorded intracortical neural activity from a human participant during attempted handwriting and reconstructed the precise trajectories of handwriting movements using a novel decoding framework. Our findings demonstrate successful handwriting trajectory reconstruction, with performance significantly surpassing traditional methods across both simulated and experimental datasets, including single‐day and multi‐day decoding scenarios. Over 52% of the decoded trajectories were recognized as legible text using generic handwriting recognition software, and by employing dynamic time warping (DTW)‐based template matching, the recognition rate reached up to 91.1% within a 1000‐character database. This innovative framework was further applied to a previously published English letter handwriting dataset,^[^ ^7^ ^]^ which had been used solely for classification purposes, demonstrating the ability to reconstruct single‐trial trajectories while achieving improved letter recognition. By addressing the misalignment issue through this novel framework, our results enable high‐performance trajectory‐based brain‐to‐text translation, holding promise for broader applications across diverse languages. This approach opens new avenues for individuals with motor deficits to communicate through written characters, regardless of linguistic complexity.

Our study introduced the DILATE loss^[^ ^20^ ^]^ in clinical BCIs, which optimally aligned neural signals and handwriting labels during the training of the regression model, and achieved superior decoding performance in both Chinese character and English letter handwriting dataset. Label misalignment with “thoughts of motion” is a prevalent issue for training an accurate decoder in clinical BCIs, especially when continuous output is required. As a contrast, conventional‐used MSE loss performs poorly with signal distortions conditions,^[^ ^20^, ^21^, ^22^, ^25^ ^]^ thus not feasible for this clinical BCI application. Alternatively, previous studies have attempted to solve the signal misalignment problem with time‐warping method,^[^ ^27^, ^28^ ^]^ which primarily align signals to a common template in an unsupervised way. However, this method does not ensure that the aligned signals correspond accurately with their kinematic labels, which is the required in our application. While a speech BCI system^[^ ^6^ ^]^ introduced connectionist temporal classification (CTC) loss^[^ ^29^ ^]^ to alleviate the problem of lack of ground truth, but this approach is limited to sequence alignment for classification tasks and cannot be extended to regression task like those in our study. Our results thus provide an optimal way to address the misalignment challenge in the regression‐based BCIs, eliminating the need to pre‐align the signal and label before decoder training.

We introduced the trajectory‐based handwriting paradigm for BCI, i.e., decoding the trajectory of attempted handwriting and then recognizing the trajectories as standard texts. Although direct classification of letters had achieved superior performance,^[^ ^7^ ^]^ trajectory decoding for handwriting offers unique advantages, including 1) generalizability to any written characters, including those not seen in training dataset; 2) naturalistic output that is more intuitive for users and enables applications beyond text (e.g., drawing); 3) accommodation to stylistic variations in handwriting, which supports personalized BCIs. A previous study showcased the trajectories of single‐letter handwritings only with trial‐averaged neural activity.^[^ ^7^ ^]^ Due to the precise alignment between neural activity and handwriting kinematics in our study, we have been able to reconstruct complex writing trajectories as human recognizable characters on a single‐trial basis. Other researches have predominantly concentrated on decoding straight movements in arm‐reach distances to control computer cursors^[^ ^3^, ^30^ ^]^ or prosthetics.^[^ ^2^, ^31^ ^]^ Preliminary studies have also explored the decoding of simple curved drawings in monkeys.^[^ ^23^, ^32^ ^]^ One critical challenge was the extraction of accurate speed profile which is nonlinearly encoded.^[^ ^33^ ^]^ Our study demonstrated the ability to reconstruct the intricate handwriting trajectory, which occurs within a significantly smaller range but encompasses distinct spatial and temporal dynamics.^[^ ^34^ ^]^

The neural feature ESA consistently demonstrated superior decoding performance in our study, aligning with previous findings in movement decoding research.^[^ ^18^, ^35^ ^]^ ESA represents a continuous signal derived from high‐pass filtering, rectification, and low‐pass filtering of raw neural data, reflecting the instantaneous measure of spike number and amplitude from neuronal populations near the electrode.^[^ ^36^ ^]^ Unlike SUA/MUA approaches that may overlook smaller neurons, ESA captures contributions from all nearby neurons, providing a more comprehensive neural activity representation.^[^ ^37^ ^]^ Notably, ESA maintains its performance advantage even when spikes are removed, suggesting it encodes valuable sub‐spike information.^[^ ^18^ ^]^ This feature combines the stability of continuous signals with the rich kinematic information of spiking activity while circumventing pitfalls associated with spike sorting and thresholding. These characteristics make ESA particularly suitable for developing robust, high‐performance BCIs.

Meanwhile, we recognized the decoded trajectories using both generic handwriting recognition software and custom template‐matching methods. Handwriting recognition and Optical Character Recognition (OCR) have reached a high level of sophistication and are widely utilized in contemporary applications.^[^ ^38^, ^39^ ^]^ However, these standard recognition techniques are not well‐suited for recognizing trajectories in our study, which are basically one‐touch‐writing trajectories that are distinct from normal writing patterns. This is one of the reasons that the recognition rate using this method is relatively low (< 53%) in our study. On the other hand, leveraging the fact that each character has its own unique speed profile, the template‐matching method we developed achieved much higher accuracy and is robust to the size of the database. This novel strategy extends the application of handwriting BCIs to encompass various written languages, be it Latin‐based or non‐Latin, as it enables the decoding of arbitrary written trajectory as it is, thereby broadening the horizons for individuals seeking enhanced communication capabilities.

The DILATE loss was also demonstrated to improve decoding performance by multi‐day data fusion. The fusion of multi‐day data to enhance the performance of the decoder has been a difficult problem in BCI due to the two opposite effects. On one hand, fusion of multi‐day data increases the amount of data to train the decoder, which could potentially enhance the performance. On the other hand, if the data are inhomogeneous, due to instability of neuronal recording or low data quality, the performance of the decoder could be deteriorated. Most of the past studies align the multi‐day data by mapping or extracting the low‐dimensional latent dynamics using methods like canonical correlation analysis,^[^ ^40^ ^]^ non‐negative matrix factorization^[^ ^41^ ^]^ or linear mapping.^[^ ^7^ ^]^ We argue that if we could align the single‐day data more accurately to enhance the data quality, the multi‐day issue would be alleviated. Our results showed that the DILATE loss effectively reduced the misalignment problem and further enhanced the decoding performance comparing with the single‐day results. For MSE loss, the two opposite effects on decoding performance canceled with each other and achieved not significant results. Thus, we developed a novel methodology with DILATE to cope with multi‐day fusion problem in BCIs.

Our study, while promising, has several limitations that warrant acknowledgment. First, we did not conduct online decoding experiments to verify the effectiveness of the DILATE loss in an online experimental scenario. Nonetheless, this approach remains a valuable starting point for constructing an initial high‐performance decoder, which can be further refined during online testing. Second, our study still considered handwriting as a 2D plane movement, rather than employing a 3D or multi‐dimensional model. Future studies could explore the integration of 3D models to account for additional dimensions in neural data, potentially further enhancing decoding performance. Third, the characters were unique across days to avoid overfitting to specific patterns, simulating real‐world scenarios where users write diverse text. However, this makes the multi‐day results confounded by the distinct characters presented for each session. Future work could include overlapping characters across days to isolate the effects of data volume from character diversity.

Through training decoders with optimal‐aligned signals, our findings could be directly applied in clinical BCIs to enhance the decoding performance. This advancement will expedite the translation of BCI research into practical human applications, broadening the scope of BCIs to include handwriting or even drawing‐related paradigms.

## 4 Experimental Section

### Participant and Surgery

The participant enrolled in this study was a right‐handed individual who had experienced a C4‐level spinal cord injury, resulting in total sensory and motor loss below the shoulders. The microelectrode implantation surgery was conducted ≈3 years after the injury when he was 72 year old and data collection for this study was at ≈2.5 years after the surgery.^[^ ^42^ ^]^ The subject provided a statement of written informed consent prior to the study. All clinical and experimental procedures received approval from the Medical Ethics Committee of the Second Affiliated Hospital of Zhejiang University and were registered in the Chinese Clinical Trial Registry (chictr.org.cn; registration number: ChiCTR2100050705).

Two 96‐channel Utah microelectrode arrays (Blackrock Microsystem, USA) were implanted into the left precentral gyrus, specifically targeting the hand “knob” area of motor cortex (Figure 1A inset). The location of implantation was identified using functional magnetic resonance imaging (fMRI) prior to surgery when the participant attempted to perform reaching and grasping movement.

### Video‐Guided Handwriting Paradigm

To guide the handwriting process for the patient, an animation video was played on the computer monitor. The video consisted of stroke‐by‐stroke writing animation of a specific character, leading by a hand with chalk (Figure 1A). The patient was asked to attempt to write the same character with chalk on a blackboard following the guidance. A typical trial started by showing the character (in dark green) on the screen (500 ms) followed by an auditory prompt of the character's pronunciation (1000 ms). After a short delay (300 ms), a sound cue was issued, and the writing animation started. The writing consisted of both strokes and pen lifts, i.e., inter‐stroke movements. The written strokes were highlighted as light green, and the pen lifts were simplified as a direct line between the end of current stroke and the start of next stroke. The duration of writing depended on the length of the character, ranging from 4 to 8 s, which is a little bit longer than normal writing speed to adapt to the patient. The speed for each character and pen lift was constant, i.e., the duration of each stroke or pen lift is proportional to their lengths.

The handwriting videos were artificially synthesized. First, the sequences of 2D coordinates for writing each character were extracted from standard font of that character using “GetData Graph Digitizer” software, where user manually placed points along each stroke following the natural writing order. Second, each segment, defined as a straight line or an approximation of a straight line before sharp inflections, was labeled as stroke or pen lift and converted the coordinates into velocity sequences. The duration for each segment was proportional to the ratio of the segment length to the total length, and the velocity profiles in *x*‐ and *y*‐direction were defined as:

(1) $$v_{x,y}\left(t\right)=\left\{\begin{array}{c} at,\;0\leq t<T/2\\ aT-at,\;T/2\leq t\leq T \end{array}\right.\;$$

where *T* represents total duration of that segment, and *a* is the scaling factor to fit the duration of the segment. Lastly, the handwriting animations were created frame‐by‐frame according to the velocity profile above using MATLAB. The video had a black background, and there was a static dark trace of the entire character before the actual writing starts. The strokes were represented by light green lines with thick width over the static dark characters (Figure 1B). The position and velocity data used for decoding were 5‐point smoothed version of the actual traces (sampled at 20 Hz), which resembled a bell‐shaped profile (Figure 1C).

### Data collection Sessions

Neural data were recorded when the subject attempted to write various characters during 1–2 h sessions on scheduled days. During the experimental sessions, the patient was seated in a wheelchair with hands resting on a table. A computer monitor was setup in front of the patient for task visualization. Two cables were connected from the patient's head connectors to the NeuroPort data acquisition system (Blackrock Microsystem, USA), which recorded both neural signals and task timings (through serial port) simultaneously. The character dataset used in this study included 180 unique Chinese characters distributed in 6 sessions (30 characters per session, three repeats per character) across 18 days.

### Neural Signal Preprocessing

Neural signals from each channel were amplified, filtered (0.3–7500 Hz), and digitized at a sample rate of 30 kHz using NeuroPort (Blackrock Microsystem, USA). Various signal features were then extracted, including:

1. Single‐unit activity (SUA), which was extracted online after further filtering (250–5000 Hz) with a threshold of −6.25 times root mean square (rms). Single units were isolated offline using Offline Sorter (Plexon, USA).
2. Multi‐unit activity (MUA), which was extracted offline from the further filtered data (250–5000 Hz) using different threshold at −4.5× or −6.25×rms. No further spike sorting was applied.
3. Local filed potential (LFP), which was obtained by low‐passing (below 500 Hz) of raw signal and down sampled to 2000 Hz. To reduce sporadic outliers, extremes exceeding ±3 times the standard deviation from the mean were clipped, followed by a third‐order Butterworth lowpass filter. Then the mean powers for each frequency band (1–4, 3–10, 12–23, 27–38, 50–300 Hz) were calculated as signal features.
4. Local motor potential (LMP), which was the moving average of LFP in non‐overlapping 50 ms windows.^[^ ^26^ ^]^
5. Entire spiking activity (ESA), which was obtained by applying a first‐order Butterworth high‐pass filter (300 Hz) on raw signal, rectifying by taking the absolute value, first‐order Butterworth low‐pass filtering (12 Hz), and finally down‐sampling to 1 kHz.^[^ ^18^ ^]^
6. Spiking‐band power (SBP), which was obtained by applying a second‐order Butterworth bandpass filter (300–1000 Hz) to the raw signal, rectifying by taking its absolute value, and finally down‐sampled to 2 kHz.^[^ ^43^ ^]^
7. Continuous multiunit activity (cMUA), which was obtained by applying third‐order Butterworth bandpass filtering (300–6000 Hz) to raw signal, squared, low‐pass filtering using a third‐order Butterworth filter (100 Hz), clipping negative values, square rooted, and finally down‐sampled to 1 kHz.^[^ ^44^ ^]^ It was found that cMUA had a high correlation coefficient (above 0.87) and similar decoding results with ESA, thus was not used for further analysis.

To determine the precise timing of attempted handwriting relative to animation onset, principal component analysis (PCA) was conducted, which revealed significant neural activity changes in PC1 and PC2 ≈300 ms after the cue. This latency aligns with the temporal resolution of movement onset after cue (≈200–500 ms). Neural signal bin sizes (50‐400 ms) were evaluated through decoding analysis, finding that a 200 ms window provided optimal performance. Consequently, All neural signal features were processed using overlapping 200 ms bins with the 300 ms temporal shift to ensure proper alignment with handwriting kinematics. This setting is compatible with real‐time deployment which could use the 200 ms data before the current time point to output minimal latency decoding result.

### Trajectory Fitting Models

To fit the trajectory of the attempted handwriting movement from the neural signal features, Kalman Filter (KF) and long short‐term memory (LSTM) were utilized as decoder.^[^ ^18^ ^]^ The decoder input dimension is fixed at 96 for all neural features except SUA, which varies according to the number of sorted single units in each session. The decoder output consistently maintains 2D, representing either velocity or position coordinates in *x‐* and *y‐*axis. The KF uses linear system state equation and the input‐output data observed to estimate the system's state optimally. The KF employs a recursive approach for state prediction and state updates as follows:

(2) $${\hat{x}}_{k}^{-}=A{\hat{x}}_{k-1}^{-}+Bu_{k}$$

(3) $$P_{k}^{-}=AP_{k-1}A^{T}+Q$$

(4) $$K_{k}=P_{k}^{-}\;H^{T}{\left(HP_{k}^{-}H^{T}+R\right)}^{-1}$$

(5) $${\hat{x}}_{k}={\hat{x}}_{k}^{-}+K_{k}\left(z_{k}-H{\hat{x}}_{k}^{-}\right)$$

(6) $$P_{k}=\left(I-K_{k}H\right)P_{k}^{-}$$

where ${\hat{x}}_{k}^{-}$ is the predicted state value, ${\hat{x}}_{k}$ is the optimal estimate of the state, *A* is the state transition matrix, *B* is the control input matrix, *H* is the state observation matrix, *Q* and *R* represent the covariances, which respectively characterize the deviations of the state values and observation values.

The LSTM is a type of recurrent neural network (RNN) designed specifically to solve the issue of long‐term dependencies in traditional RNNs. The core of LSTM is the cell state, which serves to stably preserve long‐term memory in the model. LSTM utilizes gate mechanisms to control the removal or addition of information to the cell state. The forget gate determines which information should be discarded from the cell state, the input gate determines which new information should be added to the cell state, and the output gate determines the features of the cell state to be outputted. The description is as follows:

(7) $$f_{t}=\sigma \left(W_{f}x_{t}+U_{f}h_{t-1}+b_{f}\right)$$

(8) $$i_{t}=\sigma \left(W_{i}x_{t}+U_{i}h_{t-1}+b_{i}\right)$$

(9) $${\tilde{c}}_{t}=\tanh\left(W_{c}x_{t}+U_{c}h_{t-1}+b_{c}\right)$$

(10) $$o_{t}=\sigma \left(W_{o}x_{t}+U_{o}h_{t-1}+b_{o}\right)$$

(11) $$c_{t}=f_{t}\;⊙c_{t-1}+i_{t}⊙{\tilde{c}}_{t}$$

(12) $$h_{t}=o_{t}⊙\;\tanh\left(c_{t}\right)$$

where *x* represents the input, *h* represents the output, *f* represents the forget gate, *i* represents the input gate, *o* represents the output gate, *c* represents the cell memory, *tanh* denotes the hyperbolic tangent activation function. The symbols σ and ⊙ represent the sigmoid activation function and element‐wise multiplication operator. The number of units in the LSTM was 512 and the network was trained with batch size of 1, dropout rate of 0 and learning rate of 0.001.

In addition to LSTM, the decoding performance of four alternative architectures was evaluated (Figure S4, Supporting Information): Simple Recurrent Neural Networks (SRNN), Gated Recurrent Units (GRU),^[^ ^45^ ^]^ Temporal Convolutional Networks (TCN),^[^ ^46^ ^]^ and Transformers.^[^ ^47^ ^]^ each architecture and its implementation in the study are described below.

The SRNN represents a classical approach for processing variable‐length sequences through recurrent connections. Its core mechanism involves propagating hidden states across time steps according to:

(13) $$h_{t}=\;\tanh\left(Wx_{t}+Uh_{t-1}+b_{h}\right)$$

The symbols and parameters are consistent with the LSTM implementation.

GRU improves upon traditional RNNs by addressing long‐range dependency and gradient instability issues through simplified gating mechanisms (reset and update gates):

(14) $$z_{t}=\sigma \left(W_{z}x_{t}+U_{z}h_{t-1}+b_{z}\right)$$

(15) $$r_{t}=\sigma \left(W_{r}x_{t}+U_{r}h_{t-1}+b_{r}\right)$$

(16) $${\tilde{h}}_{t}=\;\tanh\left(W_{h}x_{t}+U_{c}\left(r_{t}\;⊙h_{t-1}\right)+b_{h}\right)$$

(17) $$h_{t}=\left(1-\;z_{t}\right)⊙h_{t-1}+z_{t}⊙{\tilde{h}}_{t}$$

where *z* and *r* represent the update and reset gates respectively, with other parameters maintaining consistency with the LSTM and SRNN implementations.

TCNs employ causal and dilated convolutions to capture long‐term dependencies in temporal data, supplemented by residual connections to mitigate gradient issues in deep networks. For a 1D sequence input x ∈ ℝ^n^ and filter f: {0,…,k‐1} → ℝ, the dilated convolution operation F at sequence position s is:

(18) $$F\left(s\right)=\left(x{*}_{d}f\right)\left(s\right)=\sum_{i=0}^{k-1}f\left(i\right)\cdot x_{s-d\cdot i}\;$$

where *d* is the exponentially increasing dilation factor, *k* is the filter size, and *s*‐*d*·*i* ensures temporal causality. Each residual block implements:

(19) $$o\;=\;\mathrm{Activation}\left(x+F\left(x\right)\right)$$

Transformer utilizes self‐attention mechanisms rather than recurrent or convolutional operations, which is good at capturing long‐distance dependencies. only the encoder component of the Transformer model is used for decoding. This component is described as:

(20) $$X^{\prime }=X+\mathrm{PE}$$

(21) $$q\;=W_{q}\;X^{\prime },\;k\;=W_{k}\;X^{\prime },\;v\;=W_{v}\;X^{\prime }$$

(22) $$\;q_{i}={\;\mathrm{qW}}_{i}^{q},\;\;k_{i}={\;\mathrm{kW}}_{i}^{k},\;\;v_{i}={\;\mathrm{vW}}_{i}^{v},\;i\;=1,\text{…},n$$

(23) $$\mathrm{hea}d_{i}=\;\mathrm{Attention}\left(q_{i},\;k_{i},\;v_{i}\right)=\;\mathrm{softmax}\left(\frac{q_{i}k_{i}^{T}}{\sqrt{d_{k}}}\right)v_{i}$$

(24) $$\mathrm{MultiHead}\left(X^{\prime }\right)=\;\mathrm{Concat}\left(\mathrm{hea}d_{1},\text{…},\mathrm{hea}d_{n}\right)W_{o}$$

(25) $$Y\;=\;\mathrm{LayerNorm}\left(X^{\prime }+\mathrm{MultiHead}\left(X^{\prime }\right)\right)$$

(26) $$\mathrm{FFN}\left(Y\right)=\;\mathrm{ReLU}\left(YW_{1}+b_{1}\right)W_{2}+b_{2}$$

(27) $$Z\;=\;\mathrm{LayerNorm}\left(Y+\mathrm{FFN}\left(Y\right)\right)$$

where *PE* is the position encoding, which labels the position according to the channel and time using the sine‐cosine function. *Concat* denotes the splicing operation. *LayerNorm* denotes the layer normalization operation. *ReLU* denotes the rectified linear unit.

All RNN variants (SRNN, GRU, LSTM) maintain identical layer counts and unit sizes. For TCN and Transformer models, hyperparameters were adjusted to achieve comparable parameter counts with the LSTM baseline. Complete training hyperparameters are provided in Table S1 (Supporting Information). Each architecture was appended with an exponential linear unit (ELU) activation function followed by a fully‐connected linear layer to construct the complete network model.

The performance of decoding models was evaluated using leave‐one‐character‐out cross‐validation througout the paper, where all repetitions of the held‐out character were excluded from the training set. For single‐day decoding, each cross‐validation round used 29 characters (3 repetitions/character, 87 samples in total) for training and the remaining single character for testing. Both velocity and position of the handwriting were used to decode the trajectory of characters. For velocity model, an additional step that integrating velocity along the path was calculated to reconstruct the position, i.e., trajectory (Figure S1A, Supporting Information). Finally, Mean Squared Error (MSE) and Pearson's Correlation Coefficient (CC) were used as evaluation metrics for decoding performance and paired Wilcoxon signed‐rank tests to assess statistical differences in decoding performance between different features and decoding methods.

To mitigate network overfitting during training, neural signal features were augmented by adding Gaussian noise with standard deviation proportional to each channel's feature variability (scaling factors of 0.2, 0.4, 0.6, 0.8, 1.0) (Figure S6, Supporting Information). The impact of noise injection was systematically evaluated by analyzing both the training loss values and the DTW‐based recognition accuracy across different noise levels.

### Trajectory Recognition

Decoded handwriting trajectories were recognized as text in two ways. First, the trajectory for each character was fed into an online generic handwriting recognition software through their APIs (teshuzi.com, v1, available at https://rapidapi.com/jackzhu652‐o7wnlo8g_MO/api/handwriting‐recognition1). The first Chinese character output by the algorithm, which has the highest similarity score, was selected as the recognition outcome. Second, the decoded trajectories were recognized by matching them against a database of velocity profiles from standard characters. To accomplish this, trajectories for up to 1000 commonly used characters were extracted (using methods above) and converted them into their corresponding velocity profiles. To evaluate robustness to inter‐user variability, the velocity profiles for the 180 characters in the library were generated by a different user than those used for the experimental prompts. While this approach preserved identical spatial paths, it introduced variations in temporal dynamics. Dynamic Time Warping (DTW) and correlation coefficient (CC) were employed to quantify the similarity between the decoded velocity and velocity profiles in the library. DTW permits temporal stretch and delay, thereby enabling a more precise capture of the inter‐sequence similarity (Figure S2B,C, Supporting Information). Given the O(n^2^) computational complexity of standard DTW, the fastDTW algorithm^[^ ^48^ ^]^ was implemented to efficiently compute DTW distances. By constraining the search space to a neighborhood around the projected path from the lower resolution, fastDTW reduces the computational complexity to O(n) while maintaining alignment accuracy, as the path length scales linearly with input size. The character with the highest similarity score, as determined by DTW or CC, was selected as the final recognition result.

For two *d*‐dimensional time series *y* and *z* of length *m* and *n*, the DTW finds an optimal alignment path *A* ⊂ {0, 1}*^m×n^*, where *A_i, j_* = 1 when *y_i_* and *z_j_* are aligned. In the set of admissible alignment paths *A_m, n_*, the alignment paths are always connected to (*m*, *n*) from (1, 1) and when *A_i, j_* = 1, the next alignment path point can only be *A_i+1, j_*, *A_i, j+1_* or *A_i+1, j+1_*. *Δ*(*y*, *z*) is the point‐to‐point loss matrix for the sequences *y* and *z*, where the point‐to‐point loss is computed using the Euclidean distance, i.e., *Δ(y, z)_i, j_* = ${\left\|y_{i}-z_{j}\right\|}_{2}^{2}$. The DTW computes the alignment path with the smallest cumulative cost, that is

(28) $$\mathrm{DTW}\left(y,\;z\right)=\min_{A\subset \mathrm{Am},\;n}\langle A,\;\Delta \left(y,\;z\right)\rangle \;$$

where 〈 ·,   · 〉 denotes the inner product of two matrices. In practice, DTW usually calculates the cumulative distance of the optimally alignment paths using a dynamic programming approach with the following recursive formula:

(29) $$\gamma \left(i,\;j\right)=\;d\left(y_{i},\;z_{j}\right)+\min\left(\gamma \left(i-1,\;j\right),\;\gamma \left(i-1,\;j-1\right),\;\gamma \left(i,\;j-1\right)\right)$$

where the cumulative distance *γ*(*i*,*j*) is the sum of the distance *d(i,j)* of the current alignment pair, i.e., the distance between points *y_i_* and *z_j_*. This formulation efficiently determines the global minimum‐distance alignment path by systematically evaluating all possible warping paths while satisfying temporal ordering constraints. Using the optimal alignment path determined by DTW, the predicted sequence was temporally aligned with the ground truth labels. The DTW formulation ensures that for each time index *i* in the reference sequence, there exists at least one corresponding aligned index *j* in the predicted sequence. The warping transformation is computed as

(30) $$\mathrm{inde}x_{i}=\;\min\left\{j|\;A_{i,\;j}=1\right\}$$

(31) $$\mathrm{aligned}\;z\;=\left\{z_{\mathrm{inde}x_{1}},z_{\mathrm{inde}x_{2}},\;\text{…},z_{\mathrm{inde}x_{m}}\right\}$$

Recognition rates were computed across library sizes ranging from 180 to 1000 characters. For each size (except for 180 and 1000), a subset of characters was randomly (while ensuring inclusion of all the 180 characters used in the experiment) and repeated this sampling 1000 times to obtain the mean recognition rate and standard deviation. To characterize recognition errors, the confusion matrix from DTW‐based recognition was analyzed by examining shared features among misclassified characters (Figure S7, Supporting Information). For all character pairs, 1) mean similarity metrics (correlation coefficient, stroke count difference, and DTW distance) were computed, and 2) averages for the misclassified pairs (non‐diagonal confusion matrix elements). These distributions were then statistically compared to identify significant differences between randomly and incorrectly matched characters. Statistical comparisons between decoding schemes were performed using the Mann‐Whitney U‐test.

### DILATE Loss

The DILATE loss was based on smooth approximation of DTW with a temporal term.^[^ ^20^ ^]^ To deal with the non‐differentiability of DTW, the shape loss part of DILATE introduces a differentiable minimization function that replaces the hard minimization operation in DTW, so that the loss function gains global differentiability. The soft minimum operation and shape loss are as follows:

(32) $$\mathrm{softmi}n_{\gamma }\left(a_{1},\;\text{…},\;a_{n}\right)=-\gamma \log\left(\sum_{i}^{n}\exp\left(-a_{i}/\gamma \right)\right)$$

(33) $$\mathrm{softDT}W_{\gamma }\left(y,\;z\right)=-\gamma \log\left(\sum_{A\subset \mathrm{Am},\;n}\exp\left(-\langle A,\;\Delta \left(y,\;z\right)\rangle /\gamma \right)\right)$$

To make the model not only focus on the similarity of sequence shapes when predicting, but also consider the time accuracy of the prediction results, DILATE adds a time loss function along with softDTW. This function was an improved version of the Temporal Distortion Index (TDI), which was a common time similarity. The TDI is defined as

(34) $$\mathrm{TDI}\left(y,z\right)=\langle A^{*},\;\Omega_{\mathrm{dissim}}\rangle \;$$

where $A^{*}=\underset{A\subset \mathrm{Am},\;n}{\mathrm{argmin}}\left\langle A,\;\Delta (y,\;z)\right\rangle$ is the DTW optimal path, Ω_dissim_ is a matrix of size *m*×*n*, where Ω_dissim_(i, j)  = (i − j)^2^ , serves to penalize alignment point pairs that deviate from the diagonal of the path matrix.

The original version of TDI is non‐differentiable because the optimal path A is non‐differentiable with respect to Δ. To deal with this problem, the time‐loss part of DILATE handles this using a relaxed optimal path $A_{\gamma }^{*}$, which is defined as the gradient of the softDTW_γ_. The relaxed optimal path $A_{\gamma }^{*}$ and time loss are as follows:

(35) $$A_{\gamma }^{*}=\nabla_{\Delta }\;\mathrm{softDT}W_{\gamma }\left(y,\;z\right)=\frac{\sum_{A\subset \mathrm{Am},\;n}\mathrm{Aexp}\left(-\langle A,\;\Delta \left(y,\;z\right)\rangle /\gamma \right)}{\sum_{A\subset \mathrm{Am},\;n}\exp\left(-\langle A,\;\Delta \left(y,\;z\right)\rangle /\gamma \right)}\;$$

(36) $$\mathrm{softTD}I_{\gamma }\left(y,z\right)=\langle A^{*},\;\Omega_{\mathrm{dissim}}\rangle =\frac{\sum_{A\subset \mathrm{Am},\;n}\langle A,\;\Omega_{\mathrm{dissim}}\rangle \exp\left(-\langle A,\;\Delta \left(y,\;z\right)\rangle /\gamma \right)}{\sum_{A\subset \mathrm{Am},\;n}\exp\left(-\langle A,\;\Delta \left(y,\;z\right)\rangle /\gamma \right)}\;$$

Ultimately, the DILATE loss is weighted sum of the shape loss and the time loss, that is

(37) $$\mathrm{DILAT}E_{\alpha ,\gamma }\left(y,z\right)=\alpha \cdot \mathrm{softDT}W_{\gamma }\left(y,\;z\right)+\left(1-\alpha \right)\cdot \mathrm{softTD}I_{\gamma }\left(y,z\right)$$

### DILATE Simulation

To test the performance advantage of using DILATE over MSE loss, a simulation experiment was designed with the following steps. Two datasets, the 180 Chinese characters in this study and the 26 English letters,^[^ ^7^ ^]^ were tested separately.

1) Reconstruct the prompted velocity sequence *V* into a trajectory sequence *P* and assume that the velocity between two points of the trajectory sequence is uniform.

2) Randomly sample time points from a uniform distribution and sort them, with values ranging from 0 to the time step of the trajectory sequence, and the number of values is the time step of the trajectory sequence, calculate the positions of the corresponding time points in the trajectory sequence to obtain the resampled trajectory sequence *P'*, and differentiate to obtain the simulated velocity sequence *V'*. The resampling time points are random and different for different simulated trials.

3) A fixed linear layer neural network is used as an encoder with input *V'* to generate the simulated neural signal *S'*.

4) Train the linear layer neural network with a 5‐fold cross‐validation scheme using two losses separately, with *S'* as the signal and *V* as the target, to obtain the prediction *Vp*.

5) Test the errors of *Vp* and *V'* using MSE, CC metrics, and test the direct recognition rate of *Vp*.

To visualize the applicable scenarios and advantages of DILATE, the simulation experiments were simplified. One‐dimensional speed was used for simulation, and assume that the prompted speed and actual speed are as in Figure S2A (Supporting Information). Since both encoder and decoder are linear layer neural networks, in fact, *Vp* = a*V' + *b*, where *a* and *b* are the weight and bias. Two kinds of loss values are computed for different *a* and *b*. The 3D loss topography was then plotted (as shown in Figure S2B,C, Supporting Information) separately and calculated the minimum value points. Comparing the difference between the two loss minima points, the advantage of DILATE loss in data and label misalignment scenarios can be obtained.

### DILATE‐Based Trajectory Fitting

To assess the efficacy of the DILATE loss in fitting handwriting trajectories, the same LSTM decoder above was employed, except that the loss function was replaced with DILATE. The model output and the labels were first z‐score normalized by dimension, and then calculated the DILATE loss values. This reduces the impact of the difference between the initial model output order of magnitude and the target on the loss calculation. The models were validated using a leave‐one‐character‐out cross validation approach as above. Additionally, The impact of the two hyperparameters—tradeoff α (ranging from 0.1 to 0.9) and smoothing *γ* (ranging from 0.0001 to 1)—in DILATE on decoding outcomes was explored with one example session. Normalized DTW, calculated as standard DTW distance divided by the length of the warping path (i.e., the number of steps taken to align) was adopted, as a quantitative metric for decoding performance, i.e., lower normalized DTW distance represents better decoding performance. Alongside this, The previously mentioned online handwriting recognition software was incorporated to objectively assess the recognition rate for trajectories decoded by the two loss functions.

Furthermore, temporal distortions of DILATE's decoding outcomes relative to the labels were quantified, by calculating DTW to ascertain optimal alignment paths A^*^ between decoding results and labels, where $A^{*}=\underset{A\subset \mathrm{Am},\;n}{\mathrm{argmin}}\left\langle A,\;\Delta (y,\;z)\right\rangle$. Then the offsets for each point across all trials were concatenated and the standard deviation (SD) of these offsets (after absolution) within each trial were calculated.

### Pseudo‐Online Experiment

In addition to offline decoding analysis, we conducted pseudo‐online decoding experiments to evaluate the temporal characteristics of the decoding pipeline (Figure S5, Supporting Information). Using an LSTM decoder with identical experimental parameters to our offline approach, we specifically measured processing times for test set decoding. The pseudo‐online implementation processed neural signals incrementally, with each time step undergoing: (1) median removal, (2) feature extraction, and (3) network inference to generate velocity predictions. We summed the computation time for each stepwise decoding operation as the time duration for decoding. Following trial completion, we performed DTW alignment between the predicted velocity sequence and standard templates in the character library, measuring recognition time per trial. Additionally, we documented the network training duration for each cross‐validation fold in the single‐day dataset analysis. These timing metrics were obtained on consumer‐grade hardware (AMD EPYC 7542, 503GB RAM, NVIDIA GeForce RTX 4090).

### Multi‐Day Data Fusion

The simulation experiment above was extended for multi‐day data fusion decoding. The amount of data in the training set were increased without changing the test set to simulate an increase in the amount from multi‐day. The increased training data were the velocities obtained from new character trajectories after random resampling, and the simulated neural signals were obtained after the same fixed encoder. The encoder for all characters was the same, but the resampling is random for each character. Finally, the decoding performance with increasing training data volume was tested. MSE, CC and recognition rate were employed as evaluation metrics for the simulation experiments.

To perform multi‐day data fusion decoding in real dataset, z‐score normalization was first applied to each day's neural feature (ESA) by channel, and then concatenated them into a single dataset for training the decoder, with either MSE or DILATE loss function. Leave‐one‐character‐out cross‐validation was maintained for multi‐day decoding, where each round used 174 characters (29 characters/session ×6 sessions, 3 repetitions/character, 522 smaples in total) for training and the remaining 6 characters (one from each session) for testing. The decoded velocities were subjected to calculate the MSE and DILATED loss respectively to evaluate the single‐day versus multi‐day decoding. Furthermore, normalized DTW, which depicts the resemblance between the decoded trajectory and the label, was calculated to evaluate the quality of character reconstruction for both loss functions. Wilcoxon signed‐rank tests were used to assess statistical differences in decoding performance.

### Decoding Trajectory for English Letters

The English handwriting dataset was from the previous study.^[^ ^7^ ^]^ In brief, the subject, with two microelectrode arrays implanted in the motor cortex, was instructed to attempt to handwrite 26 lowercase English letters following a “go” cue. The subject finished one single letter writing within a fixed interval without video guidance. The MUA activity was recorded through the two 96‐electrode intracortical arrays. The dataset also provided the label trajectory of each letter, from which speed profiles were extracted for decoding. The speed profiles were linearly interpolated to the same length as the neural activity. Fitting experiments were then performed using the same LSTM model as above with the two loss functions. The fitting models were cross‐validated using a leaving‐one‐letter‐out method.

The fitted results were first downscaled to 2D using t‐distributed stochastic neighbor embedding (t‐SNE), then clustered them using the HDBSCAN method,^[^ ^49^ ^]^ and finally evaluated the clustering results using metrics of Fowlkes–Mallows Index (FMI), Adjusted Rand index (ARI), and Adjusted Mutual Information (AMI). Next, The fitting results were randomly into a 90% training set and 10% test set and classified them using a k‐nearest neighbor (KNN) classifier. The classification experiment was repeated 100 times and statistically compared the classification accuracy (*t*‐test). Finally, the fitting performance of the two loss functions was compared using the generic recognition software.

## Conflict of Interest

The authors declare no conflict of interest.

## Supporting information



Supporting Information

## Data Availability

All data in the main text or the supplementary materials are available upon request. All the codes developed in this study are publicly available at https://github.com/Yaoyao‐Hao/handwriting‐DILATE‐decoding.
