# Supplementary material for: Decoding Handwriting Trajectories from Intracortical Brain Signals for Brain‐to‐Text Communication
Source: Adv Sci (Weinh). 2025 Jul 28;12(40):e05492. doi: 10.1002/advs.202505492 (PMC12561361; doi:10.1002/advs.202505492)
Supplement: Supplementary file 1 — Supporting Information [file ADVS-12-e05492-s001.docx]

Supporting Information

Decoding handwriting trajectories from intracortical brain signals for brain-to-text communication

Guangxiang Xu, Zebin Wang, Kedi Xu, Junming Zhu, Jianmin Zhang, Yueming Wang*, Yaoyao Hao*


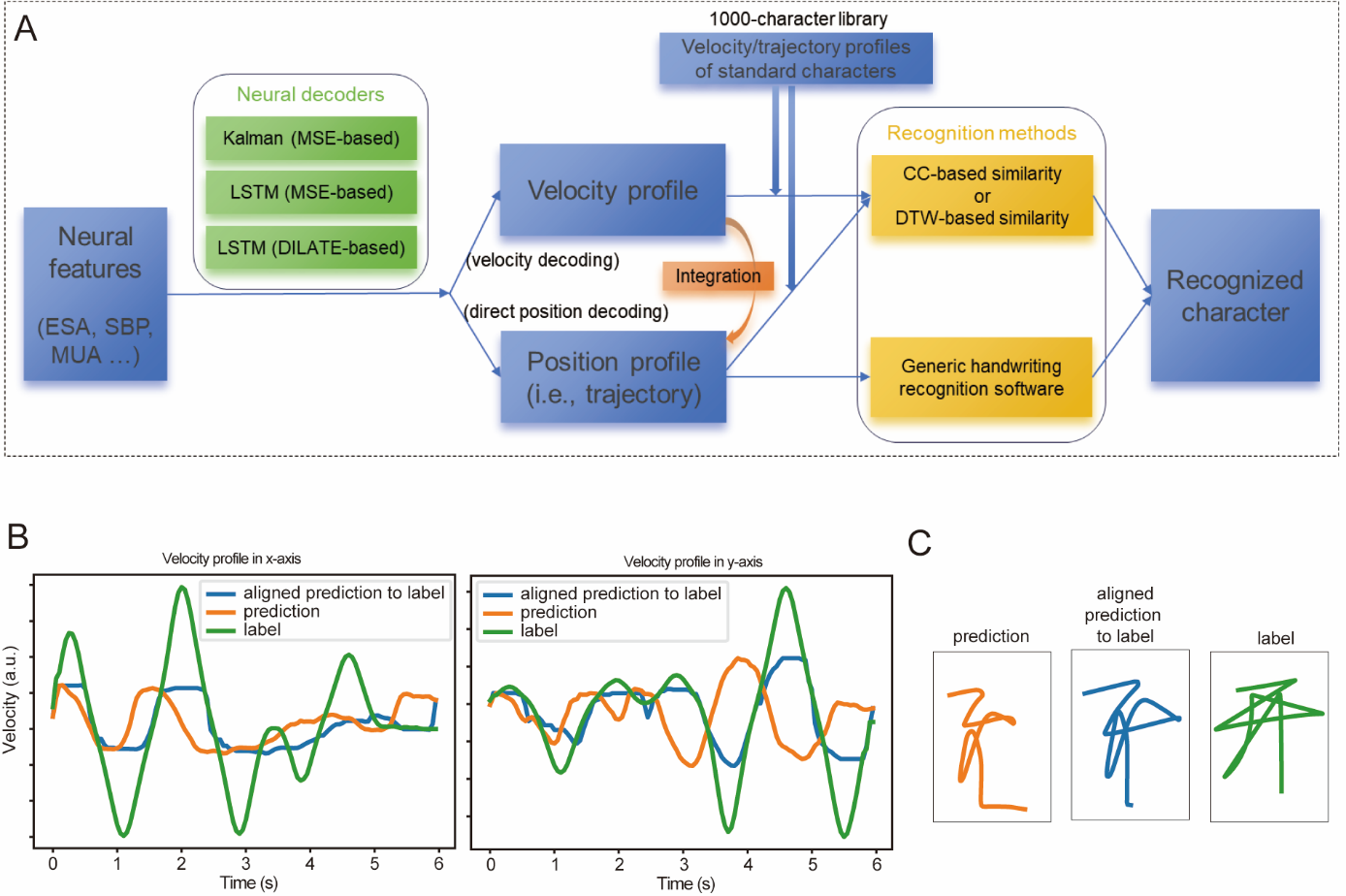


**Figure S1. Decoding flowchart and DTW warping. (A)** Flowchart of the neural decoding and recognition pipeline. The neural features were decoded as either velocity profile (then integrated as position profile) or direct position profile, using Kalman or LSTM decoders (green blocks) with either MSE or DILATE loss. The decoded profiles were recognized as standard character by either similarity measurement (CC- or DTW-based) with the 1000-character library or generic handwriting recognition software (orange blocks). **(B)** Example velocity profiles in x- and y-axis for prediction (orange), label (green), and the aligned prediction to the label (blue). **(C)** Same as (B) but for the reconstructed trajectories.


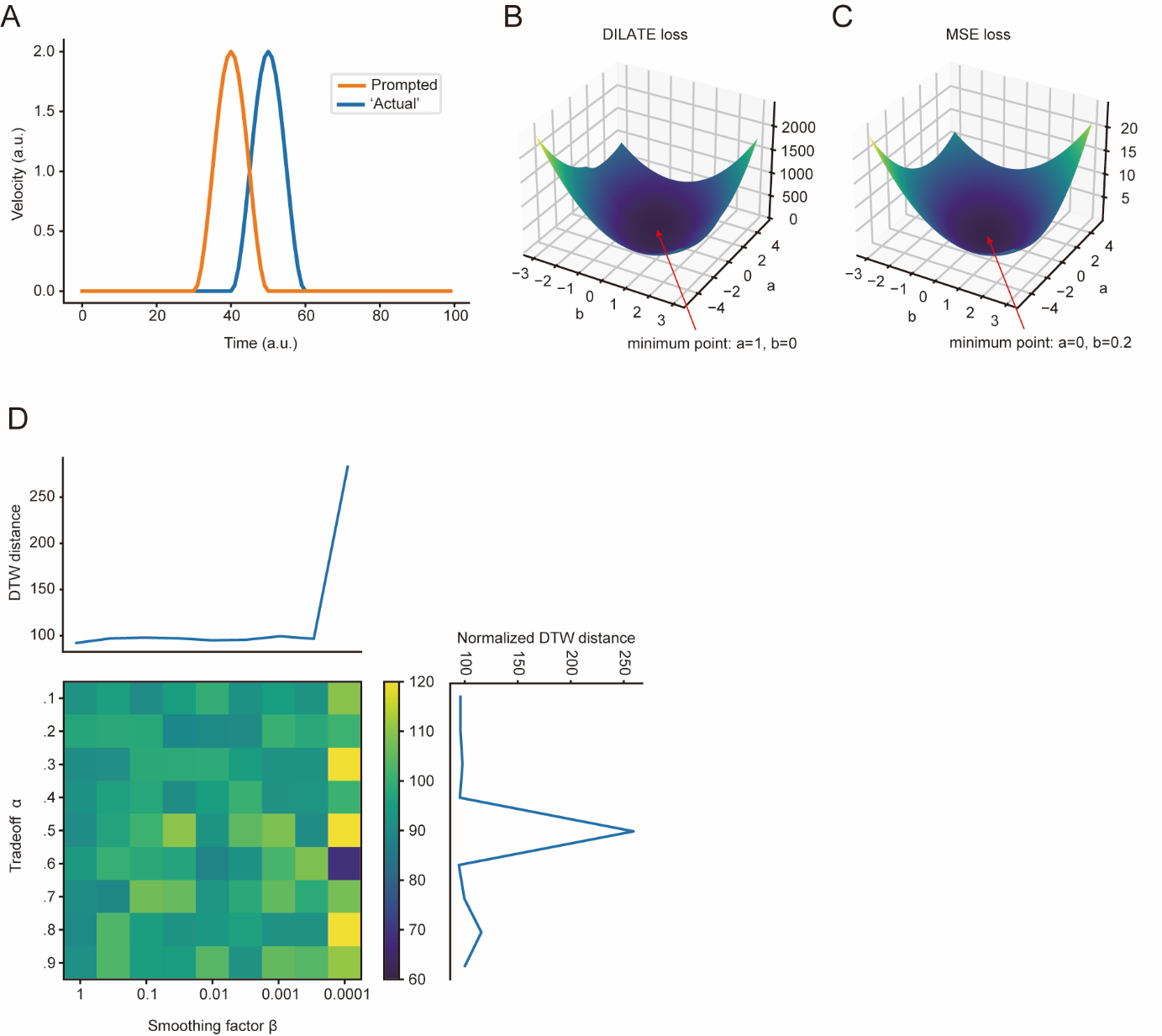


**Figure S2. DILATE loss simulation and optimization.** (**A**) Schematic representation of the prompted speed profile and the ‘actual’ profile performed by the subject. The peak of the actual velocity is 10 steps ahead relative to the prompted velocity. (**B**) 3D topographic maps of DILATE loss values between predicted and prompted velocities. The parameter *a* and *b* represent the weights and offsets of the linear mapping model *y = ax + b*. (**C**) Same as (B) but for MSE loss. **(D)** Decoding results (DTW distance) for different combinations of trade-off parameter α and smoothing parameter γ. The result was obtained from one example session.


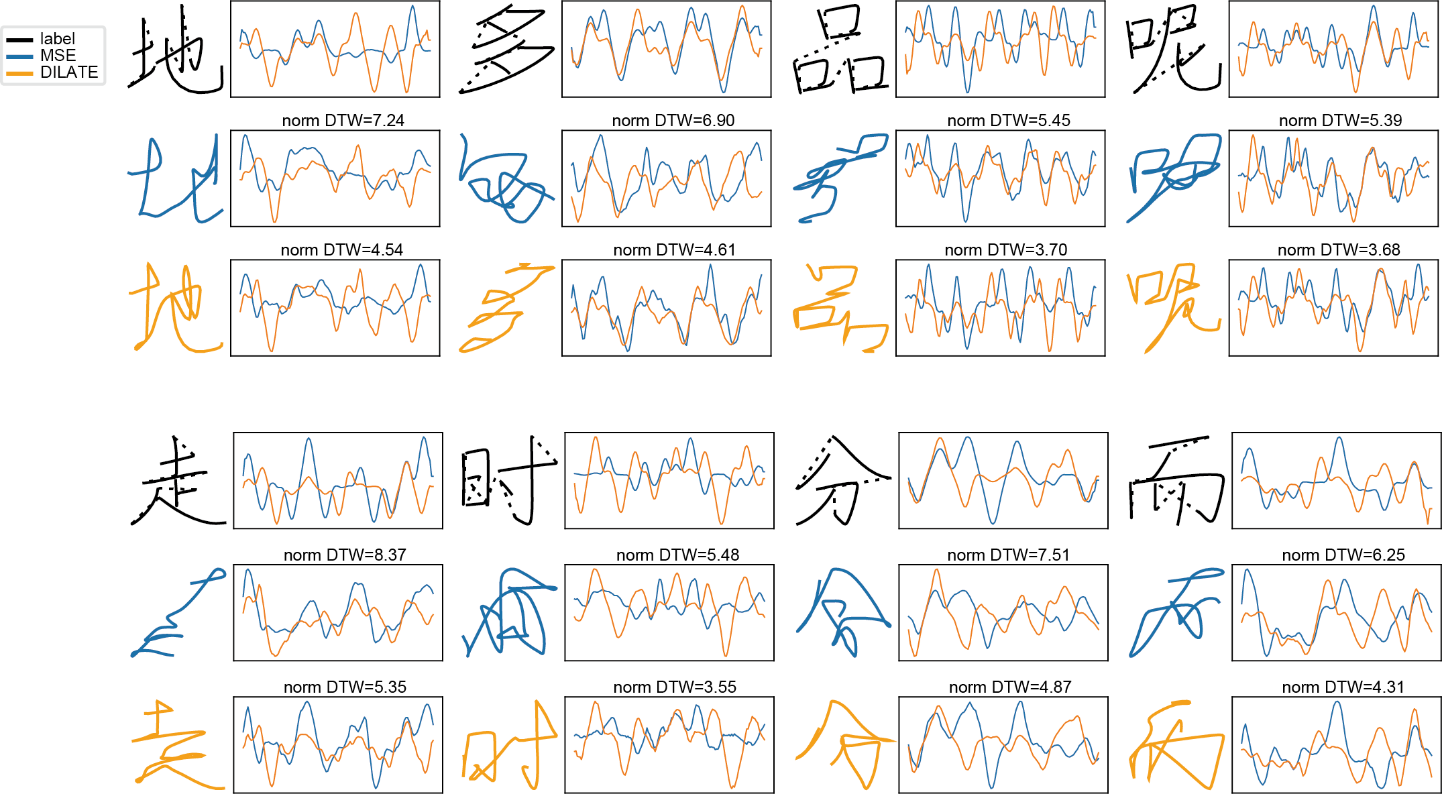


**Figure S3. Examples of handwriting trajectory decoding with MSE and DILATE loss.** The thin traces in the right side are the velocity profiles in *x-* and *y-*direction. The normalized DTW distances are labeled for each decoding.


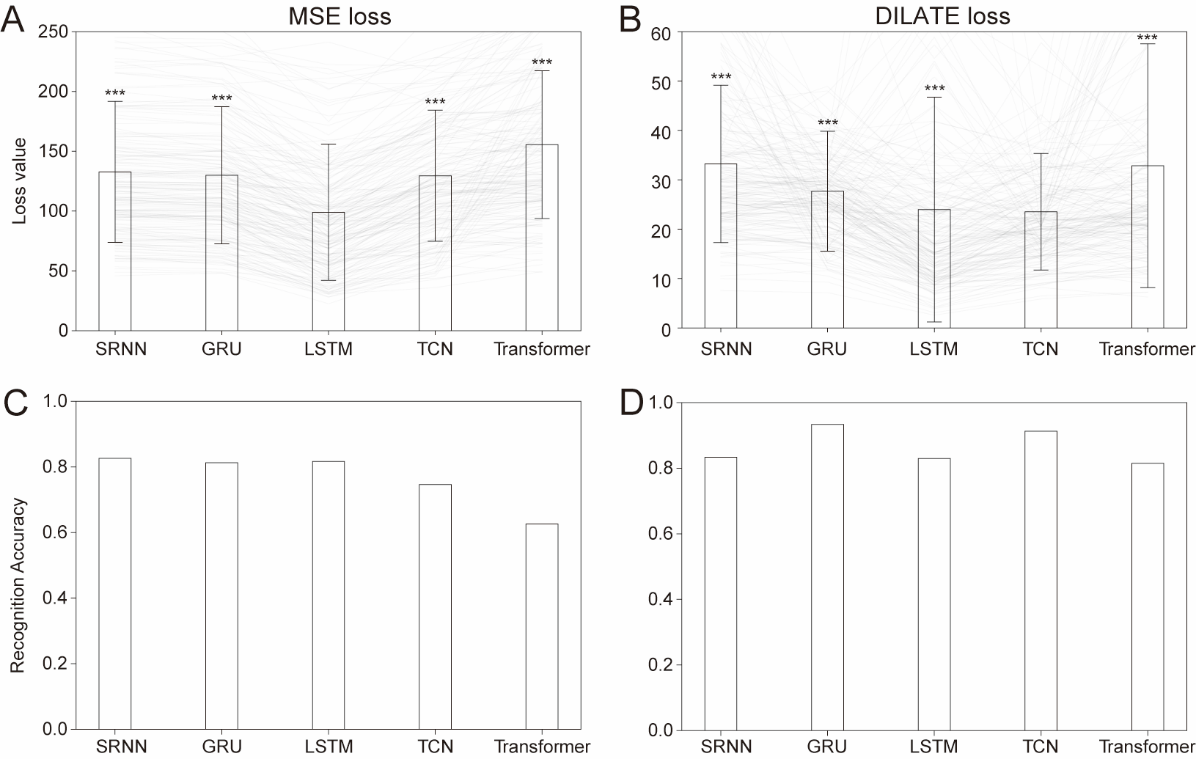


**Figure S4. Decoding with various deep learning architectures.** (**A**) The loss value of the decoders with MSE loss for various architectures (see text) for all the 180 characters tested. *** means significant difference (paired signed-rank test, *p* < 0.001). **(B)** Same as (A) but for decoders with DILATE loss. **(C)** Same as (A) but for recognition rate with DTW-based method. **(D)** Same as (C) but for decoders with DILATE loss.


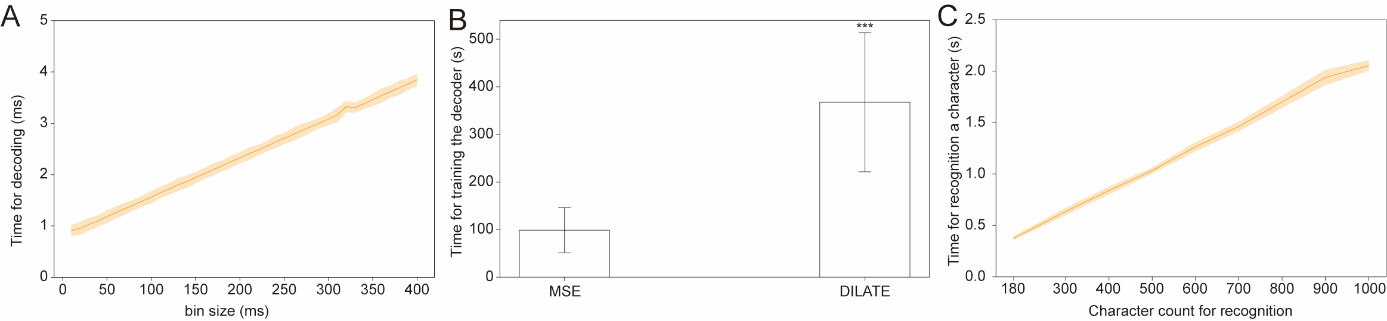


**Figure S5. Pseudo-online decoding experiments**. **(A)** The time duration for decoding as a function of bin size. The process only takes 4 ms (equivalent to 250 Hz output rate) with the longest bin size (400 ms). (B) The time duration for training the MSE and DILATE based LSTM decoder. *** means significant difference (Mann-Whitney U-test, *p* < 0.001). **(C)** The time duration for recognizing one character with DTW-based method as a function of the number of characters in the library.


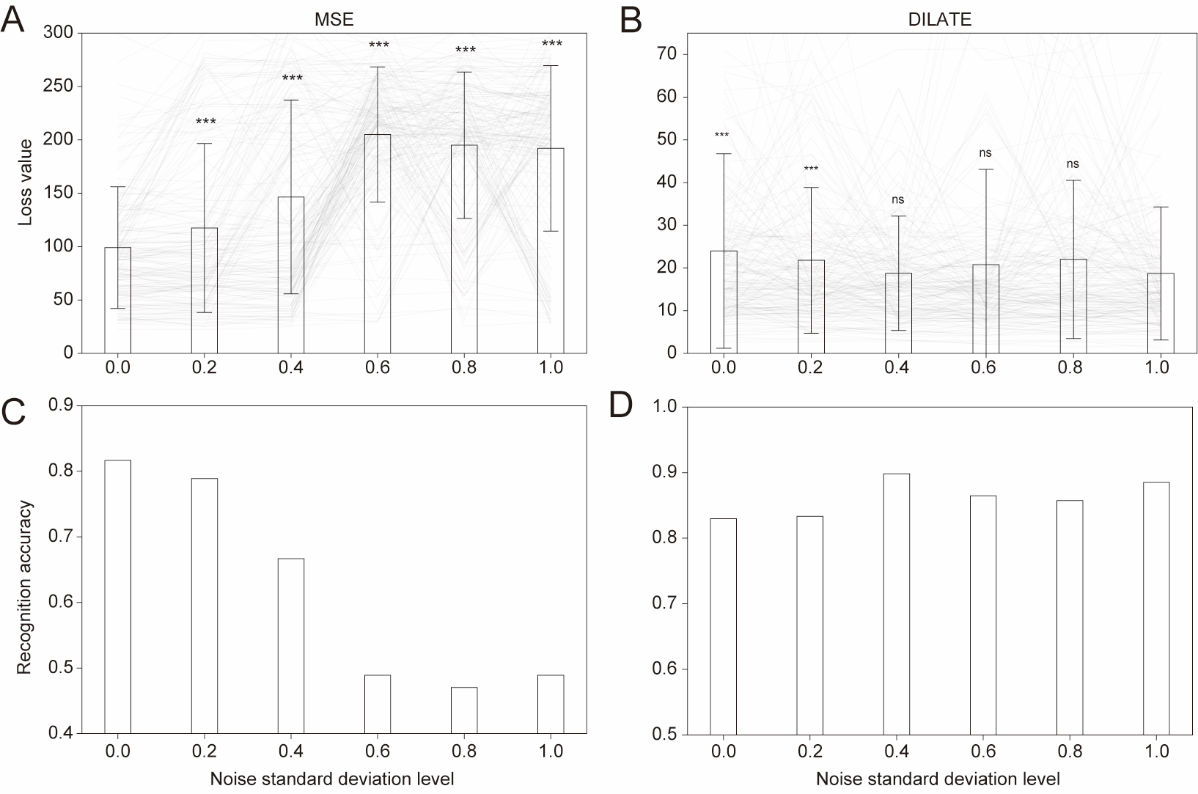


**Figure S6. Robustness of decoders to increased noise level.** **(A)** The loss value of the MSE-based decoder as a function of the Gaussian noise level, which is the relative standard deviation of the original neural feature. *** means significant difference (paired signed-rank test, *p* < 0.001). **(B)** Same as (A) but for DILATE-based decoding. **(C)** Same as (A) but for recognition rate with the DTW-based method. **(D)** Same as (C) but for DILATE-based decoding.


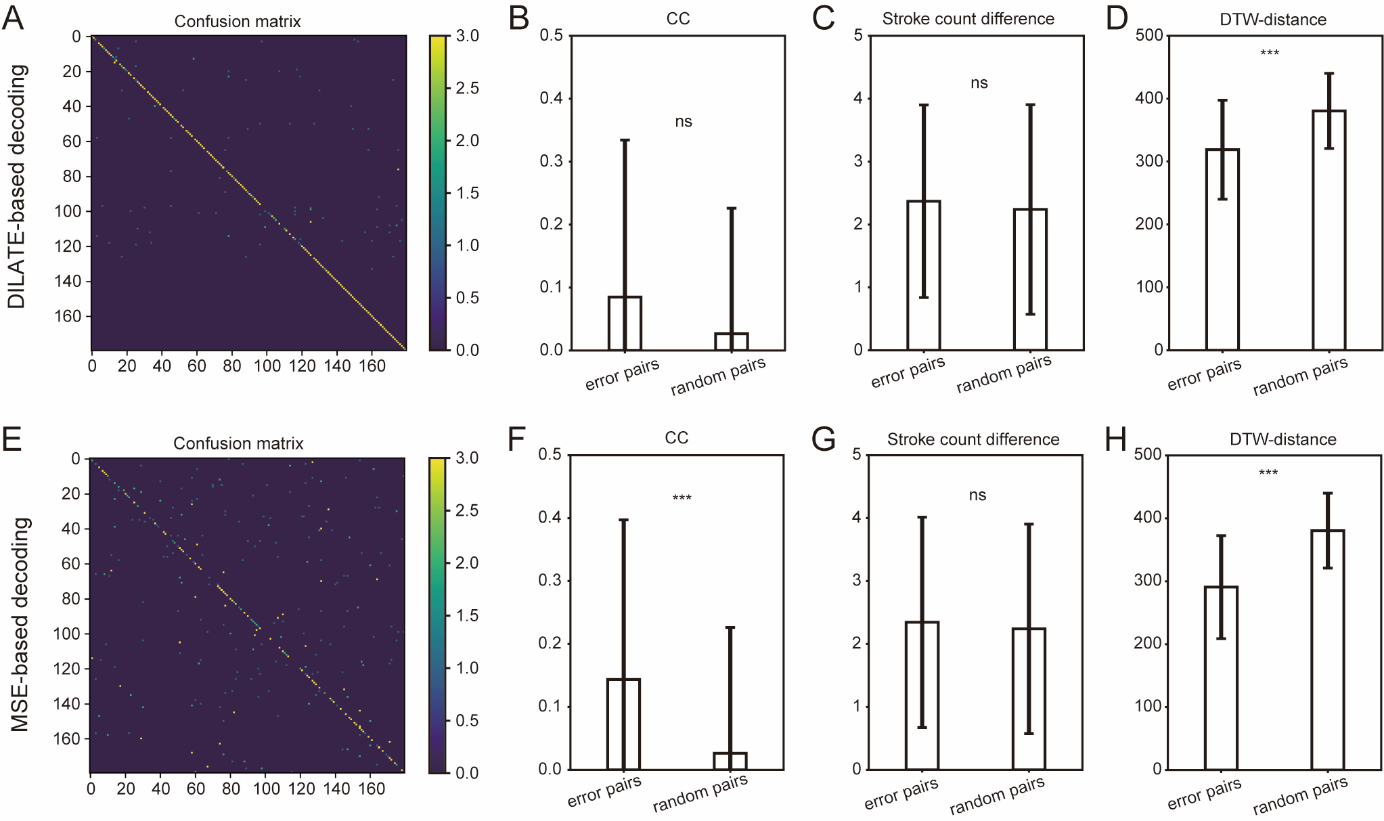


**Figure S7. Confusion matrices for DTW-based recognition and analysis.** (A) Confusion matrix for DILATE-based decoding for all the 180 characters (with 3 repeats/character). (B-D) The correlation coefficient (CC) of velocity profiles (B), stroke number difference (C) and DTW-distance of writing trajectories (D) between error or random pairs. *** means significant difference (Mann-Whitney U-test, *p* < 0.001). (E-H) Same as (A-D) but for MSE-based decoding.


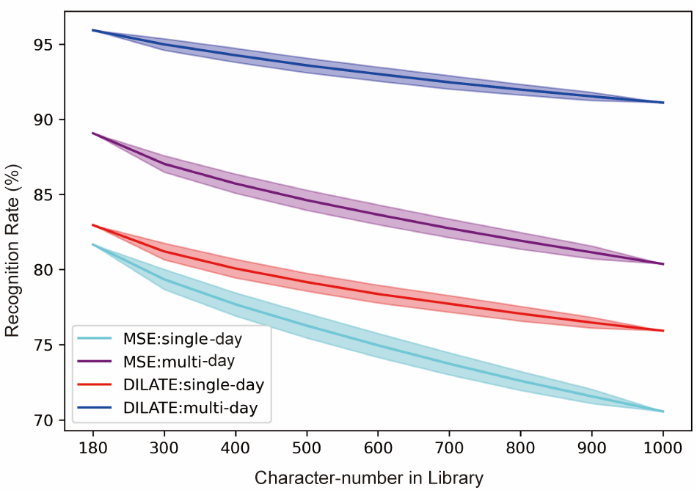


**Figure S8.** DTW-based recognition rate as a function of library size (number of characters) for combinations of different loss functions (DILATE vs. MSE) and training conditions (single-day vs. multi-day). Shaded regions represent standard deviation (SD) from 1,000 resamplings.

Table S1. Parameters for the decoding architectures.

| **Hyperparameter** | **SRNN** | **GRU** | **LSTM** |  |
| --- | --- | --- | --- | --- |
| Number of layers | 1 | 1 | 1 |  |
| Number of units | 512 | 512 | 512 |  |
| Batch size | 1 | 1 | 1 |  |
| Dropout rate | 0 | 0 | 0 |  |
| Learning rate | 0.001 | 0.001 | 0.001 |  |
| **Hyperparameter** | **LSTM** | **TCN** | **Transformer** | |
| Number of layers | 1 | 3 | 3 | |
| Kernel_size | / | 2 | / | |
| Nhead | / | / | 6 | |
| Number of all parameters | 1250306 | 1149442 | 1299170 | |
| Number of units | 512 | 512 | / | |
| Batch size | 1 | 1 | 1 | |
| Dropout rate | 0 | 0 | 0 | |
| Learning rate | 0.001 | 0.001 | 0.001 | |
